# Sub-nanomolar sensitive GZnP3 reveals TRPML1-mediated neuronal Zn^2+^ signals

**DOI:** 10.1038/s41467-019-12761-x

**Published:** 2019-10-22

**Authors:** Taylor F. Minckley, Chen Zhang, Dylan H. Fudge, Anna M. Dischler, Kate D. LeJeune, Haoxing Xu, Yan Qin

**Affiliations:** 1 Department of Biological Sciences, University of Denver, Denver, CO 80210 USA; 2 Department of Molecular, Cellular, and Developmental Biology, University of Michigan, 4114 Biological Sciences Building, 1105 North University, Ann Arbor, MI 48109 USA

**Keywords:** Biological techniques, Imaging, Molecular imaging, Neuroscience, Cellular neuroscience

## Abstract

Although numerous fluorescent Zn^2+^ sensors have been reported, it is unclear whether and how Zn^2+^ can be released from the intracellular compartments into the cytosol due to a lack of probes that can detect physiological dynamics of cytosolic Zn^2+^. Here, we create a genetically encoded sensor, GZnP3, which demonstrates unprecedented sensitivity for Zn^2+^ at sub-nanomolar concentrations. Using GZnP3 as well as GZnP3-derived vesicular targeted probes, we provide the first direct evidence that Zn^2+^ can be released from endolysosomal vesicles to the cytosol in primary hippocampal neurons through the TRPML1 channel. Such TRPML1-mediated Zn^2+^ signals are distinct from Ca^2+^ in that they are selectively present in neurons, sustain longer, and are significantly higher in neurites as compared to the soma. Together, our work not only creates highly sensitive probes for investigating sub-nanomolar Zn^2+^ dynamics, but also reveals new pools of Zn^2+^ signals that can play critical roles in neuronal function.

## Abstract (web summary)

Numerous fluorescent Zn^2+^ sensors are available but most are unsuitable to detect physiological dynamics of cytosolic Zn^2+^. In this study, the authors present a genetically encoded sensor with sub-nanomolar sensitivity and show that Zn^2 +^ is released from endolysosomal vesicles via TRPML1 in neurons.

## Introduction

Zinc is one of the most abundant trace elements and bioinformatic studies have suggested that around 2800 human proteins have Zn^2+^ coordination sites^1^. Though the majority of intracellular Zn^2+^ is in the protein-bound state, it also exists in an unbound, labile state. Cells maintain a baseline labile Zn^2+^ concentration of roughly 100 pM in the cytosol^2–5^, and a high concentration pool of labile Zn^2+^ might be stored within vesicular compartments, including lysosomes^6,7^ and synaptic vesicles^8^. It is well accepted that such vesicular Zn^2+^ in secretory cells (neurons^9^, prostate^10^, crypts of Lieberkühn^11^, pancreatic beta cells^12^) can be released extracellularly by exocytosis to execute modulatory effects between cells. However, it has been presently uncharacterized if and how these pools can be accessed within the cell.

Electrophysiological evidence found that Transient Receptor Potential Mucolipin 1 (TRPML1) is a non-selective cation channel permeable to Ca^2+^^13^, Fe^2+^^14^, Mn^2+^^15^, and Zn^2+^^14^. Mutations in TRPML1 are the genetic cause of the disease Mucolipidosis type IV (MLIV). The most prominent symptoms of MLIV are severe neurological underdevelopment and neurodegeneration. Extensive studies have focused on TRPML1-mediated Ca^2+^ signals that might have potential biological roles in lysosomal motility^16^ and autophagy^17^; however, the roles of TRPML1 in neuronal function are still unclear. Given that TRPML1 is localized on lysosomes^14,18–21^ and TRPML1 knockdown causes lysosomal Zn^2+^ accumulation^22,23^, we hypothesized that TRPML1 might mediate Zn^2+^ release from lysosomes to the cytosol.

Direct tracking of TRPML1-mediated Zn^2+^ signals requires probes that are able to monitor real time sensitive dynamic signals in living cells. The sensitivity of a biosensor is defined as the ratio of the changes in sensor output [(F−F_0_)/F_0_] to changes in Zn^2+^ concentration (Δ[Zn^2+^]), thus the ideal sensor sensitivity is dependent on the range of Zn^2+^ concentrations of interest. We predicted that opening of TRPML1 channels can release vesicular Zn^2+^ into the cytosol and might induce an elevation of local Zn^2+^ concentrations from the picomolar to nanomolar range (100 pM–1 nM). Thus we need sensors that can detect Zn^2+^ with sub-nanomolar sensitivity.

To assess sensor sensitivity, we take into account the sensor’s apparent dissociation constant (K_d_) for Zn^2+^, in situ dynamic range (maximum to minimum signal ratio), and in situ maximal sensor response above baseline. When Zn^2+^ concentration changes within 10-fold below and above the K_d_, the sensor demonstrates the most significant changes^24^. As such, we defined a sensor’s sensitive range as the concentration from 10% to 10-fold of K_d_. Most small molecule sensors possess high K_d_, limiting their sensitivity for Zn^2+^ changes between 100 pM and 1 nM^25–28^. For example, the widely utilized small molecule sensor FluoZin-3 (Thermofisher) presents a large dynamic range but a K_d_ of 15 nM, confining its sensitive detection to Zn^2+^ concentrations within the nanomolar range (~1.5–150 nM). For all previously published genetically encoded Zn^2+^ sensors, eight sensors display compatible binding affinity in the sub-nanomolar range, but their sensitivities suffer from low dynamic range (Table 1). For all the FRET sensors, their maximal response to Zn^2+^ saturation yields small signals (0–0.65) above or below the baseline in cells (Table 1), preventing them from sensitively detecting the changes in Zn^2+^ concentrations at sub-nanomolar to nanomolar levels.

**Table 1:** Comparison of GZnP3 with previously published genetically encoded Zn^2+^ sensors

Sensor type	Zn^2+^ sensor	Apparent dissociation constant (*K*_d_)	In situ dynamic range (S_max_/S_min_)	Maximal response above baseline [(S_Zn_-S_0_)/S_0_]	Ref
FRET	ZapCY1	2.5 pM^a^	4.2	0	^5^
**FRET**	**ZapCY2**	**811 pM** ^a^	**1.4**	**0.33**	^5^
FRET	ZapCmR1.1	n.d.	1.4	0.38	^61^
FRET	ZapCmR2	n.d.	1.2	0.33	^61^
**FRET**	**ZapCV2**	**2.3 nM** ^a^	**2.2**	**0.49**	^62^
FRET	ZapCV5	0.3 μM^a^	1.5	0.36	^62^
FRET	eCALWY-1	2 pM^b^	1.6	−0.07^c^	^3^
FRET	eCALWY-2	9 pM^b^	1.6	0	^3^
FRET	eCALWY-3	45 pM^b^	1.6	**−0.14** ^**c**^	^3^
**FRET**	**eCALWY-4**	**630 pM** ^b^	**1.7**	**−0.21** ^**c**^	^3^
**FRET**	**eCALWY-5**	**1.8 nM** ^b^	**1.3**	**−0.27** ^**c**^	^3^
**FRET**	**eCALWY-6**	**2.9 nM** ^**b**^	**1.3**	**−0.2** ^**c**^	^3^
FRET	redCALWY-1	12.3 pM^b^	1.2	0	^63^
**FRET**	**redCALWY-4**	**234 pM** ^b^	**1.2**	**−0.15** ^**c**^	^63^
FRET	eZinCh-1	8.2 µM^b^	n.d.	n.d.	^3, 64^
**FRET**	**eZinCh-2**	**1.0 nM** ^b^	**2.6**	**0.65**	^27, 65^
Single FP	ZnGreen1	633 nM^a^	2.5	0	^66^
Single FP	ZnGreen2	20 μM^a^	n.a	n.d	^66^
Single FP	ZnRed	166 nM and 20 μM^a^	n.a	n.d	^28, 66^
Single FP	GZnP1	34 pM^a^	2.2	0.46	^29^
**Single FP**	**GZnP2**	**352 pM** ^a^	**4.5**	**2.5**	^2^
**Single FP**	**GZnP3**	**1.3 nM** ^a^	**11**	**4**	

Bold sensors show potential detection sensitivity between 100 pM and 1 nM. FRET sensors were measured using ratio of acceptor to donor emission (∆R/R_0_) and single FP sensors were measured using single emission (∆F/F_0_)

*S*_*max*_: sensor maximum signal. *S*_*min*_: sensor minimum signal. *S*_*Zn*_: Zn^2+^-saturated signals. *S*_*0*_: baseline cytosolic sensor signal

^a^The K_d_ was measured at pH 7.4

^b^The K_d_ was measured at pH 7.1

^c^Negative values indicate sensors with turn-off kinetics upon Zn^2+^ binding

In this manuscript, we describe the development of a novel, sensitive fluorescent protein-based Zn^2+^ probe, GZnP3, capable of detecting sub-nanomolar Zn^2+^ dynamics in live cells. Using this genetically encoded sensor, which can be targeted to various subcellular compartments, we directly demonstrate for the first time that TRPML1 is permeable to physiological concentrations of Zn^2+^, and that its activation in hippocampal neurons mediates the release of Zn^2+^ from late endosomes and lysosomes to the cytosol. Additionally, TRPML1-mediated Zn^2+^ release is unique to neurons and INS-1 cells, as compared to non-secretory mammalian cell lines. Most importantly, neurites exhibit greater (2.6-fold) Zn^2+^ signals, but reduced Ca^2+^ signals, than the soma, when TRPML1 is activated in neurons.

## Results

### Engineering GZnP3 sensor with sub-nanomolar sensitivity

In order to generate a sensor with higher sub-nanomolar sensitivity than previous genetically encoded sensors, we chose the single-fluorescent protein-based platform as previously utilized to create GZnP1 and GZnP2 in our lab (Table 1)^2,29^. The GZnP family of Zn^2+^ sensors function using a circularly permuted Green Fluorescent Protein (cpGFP) fused to the two Zn^2+^-binding finger domains of yeast *Saccharomyces cerevisiae* transcription factor Zap1^2,29^. Zn^2+^ binding can induce the conformational change of the two Zn^2+^ fingers, resulting in an increase in the fluorescent intensity of cpGFP. Using a high throughput cell lysate screening assay as previously described^2^ we identified several new sensor variants with an improved dynamic range.

**Fig. 1:**
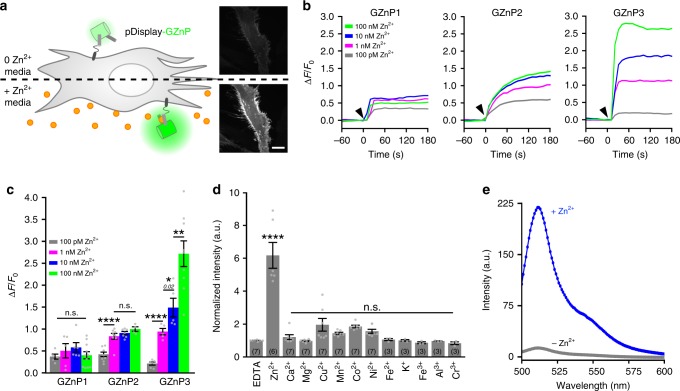
GZnP3 sensor development and characterization. **a** Schematic of the pDisplay assay showing fluorescence change of GZnP sensors in response to Zn^2+^ (left). Representative micrographs illustrated the localization of GZnP3 sensor on extracellular face of plasma membrane in HeLa cells, where GZnP3 fluorescence increased with Zn^2+^ (right). Scale bar = 20 µm. **b** Representative traces for each extracellular Zn^2+^ concentration (gray, 100 pM; green, 1 nM; blue, 10 nM; red, 100 nM) for GZnP1, GZnP2, and GZnP3. Cells were pretreated with 100 µM TPEN, washed, then imaged in the presence of 500 µM TCEP. Buffered Zn^2+^ solutions were added at 0 sec (black arrow). **c** Mean ∆F/F_0_ (± s.e.m.) at 60 s for GZnP1, GZnP2, and GZnP3. GZnP1: 100 pM (*n* = 5 cells), 1 nM (*n* = 5), 10 nM (*n* = 6), 100 nM (*n* = 11); GZnP2: 100 pM (*n* = 9), 1 nM (*n* = 8), 10 nM (*n* = 7), 100 nM (*n* = 4); GZnP3: 100 pM (*n* = 6), 1 nM (*n* = 5), 10 nM (*n* = 5), 100 nM (*n* = 9). Student’s one-tailed *t*-tests. **d** In vitro metal specificity of GZnP3 for Zn^2+^ as compared to EDTA control (12 µM), and a panel of biologically relevant divalent cations (50 µM each in the presence of 12 µM EDTA). Mean intensity ± s.e.m. (n wells displayed on bars), normalized to EDTA control. One-way ANOVA, with Dunnett’s multiple comparison to EDTA control. **e** Representative in vitro emission spectrum of GZnP3 in the apo (gray) and the Zn^2+^ saturated (blue) state when excited at 488 nm. GZnP3 displayed an emission peak at 512 nm and the peak fluorescence intensity increased 17-fold over the apo state in response to Zn^2+^. *p < 0.05, **p < 0.01, ****p < 0.0001, n.s. not significant. *p*-values displayed in italics above corresponding comparisons where applicable. Source data are provided as a Source Data file

To compare the sensor sensitivity among various GZnP variants and identify the sensor with sub-nanomolar sensitivity in live cells, we designed an in situ pDisplay assay to monitor and measure sensor responses to a range of defined picomolar to nanomolar Zn^2+^ concentrations in intact cells (Fig. 1a). GZnP sensors cloned in frame with the pDisplay vector are secreted and anchored on the extracellular side of the plasma membrane, allowing direct measurement of each sensor’s response to known Zn^2+^ concentrations. Compared to in vitro measurements with purified sensor protein, this assay provides a faster and reliable method to explore sensor sensitivity in live cells. Using HeLa cells expressing these GZnP sensor variants, we can monitor sensor response within seconds to a defined Zn^2+^ concentration (100 pM, 1 nM, 10 nM, and 100 nM) by fluorescence microscopy (Fig. 1a–c). By this pDisplay assay, we identified a new sensor GZnP3 with enhanced sensitivity to sub-nanomolar Zn^2+^. Compared to GZnP1 and GZnP2, the GZnP3 sensor clearly differentiated Zn^2+^ concentrations from 100 pM to 100 nM (Fig. 1c) and demonstrated significantly higher fluorescence change than GZnP1 and GZnP2 in response to 1 nM, 10 nM, and 100 nM Zn^2+^, separately. Such experimental results are consistent with our predictions based on K_d_ in Table 1. GZnP1 showed no sensitivity between 100 pM to 1 nM because its sensitive detection range is restricted from ~3.4–340 pM. For GZnP2, the sensitive range is ~35 pM–3.5 nM, which agrees with the pDisplay results that GZnP2 yields the highest sensitivity between 100 pM and 1 nM Zn^2+^ (Fig. 1b & c). The sensitive range of GZnP3 is similar to GZnP2, but its high dynamic range confers superior sensitivity than GZnP2. GZnP2 sensor response (ΔF/F_0_) increased from 0.42 to 0.83 in response to Zn^2+^ changing from 100 pM to 1 nM, while GZnP3 sensor increased response from 0.21 to 0.94 (Fig. 1c). Therefore, GZnP3 had a 2.25-fold greater response than GZnP2 to the same change in Zn^2+^ concentration. In addition, GZnP3 showed a faster turn-on kinetics than GZnP2 in response to Zn^2+^ (Supplementary Fig. 2b, c).

The biophysical features of GZnP3 sensors were revealed by in vitro characterization. GZnP3 showed turn-on fluorescence response to Zn^2+^ in vitro with nanomolar affinity (K_d_ = 1.3 nM) (Fig. 1e, Supplementary Fig. 2a and Supplementary Table 1). The GZnP3 sensor was specific for Zn^2+^ over other biologically relevant metals (Ca^2+^, Mg^2+^, Cu^2+^, Mn^2+^, Co^2+^, Ni^2+^, Fe^2+^, K^+^, Fe^3+^, Al^3+^, or Cr^3+^) (Fig. 1d). Metal specificity was additionally investigated in HeLa cells to examine the sensor response to Fe^2+^ and Ca^2+^ that can also be transported by TRPML1. Upon addition of ionomycin, a Ca^2+^ ionophore, in the presence of 1.26 mM extracellular Ca^2+^, there was no observed changes in GZnP3 fluorescence, confirming that Ca^2+^ does not affect baseline GZnP3 fluorescence (Supplementary Fig. 1a). Additionally, the Fe^2+^ chelator, 2,2’-bipyridyl, showed no effects on baseline GZnP3 fluorescence (Supplementary Fig. 1b) while addition of 100 µM N,N,N′,N′-tetrakis(2-pyridinylmethyl)-1,2-ethanediamine (TPEN), a Zn^2+^ chelator, showed a clear reduction of GZnP3 fluorescence. Both the quantum yield (QY) and the extinction coefficient of GZnP3 increased from the apo to Zn^2+^-bound state (Supplementary Table 1), indicating that Zn^2+^ causes increased brightness by positively affecting both photon absorption and fluorescent photon emission. GZnP3 has demonstrated a superior ability to quickly and sensitively detect in situ Zn^2+^ fluctuations across the sub-nanomolar to nanomolar range (0.1–100 nM) with specific response to Zn^2+^, thus it will be a vital tool for studying various intracellular Zn^2+^ dynamics.

### TRPML1 channel is permeable to physiological levels of Zn^2+^

**Fig. 2:**
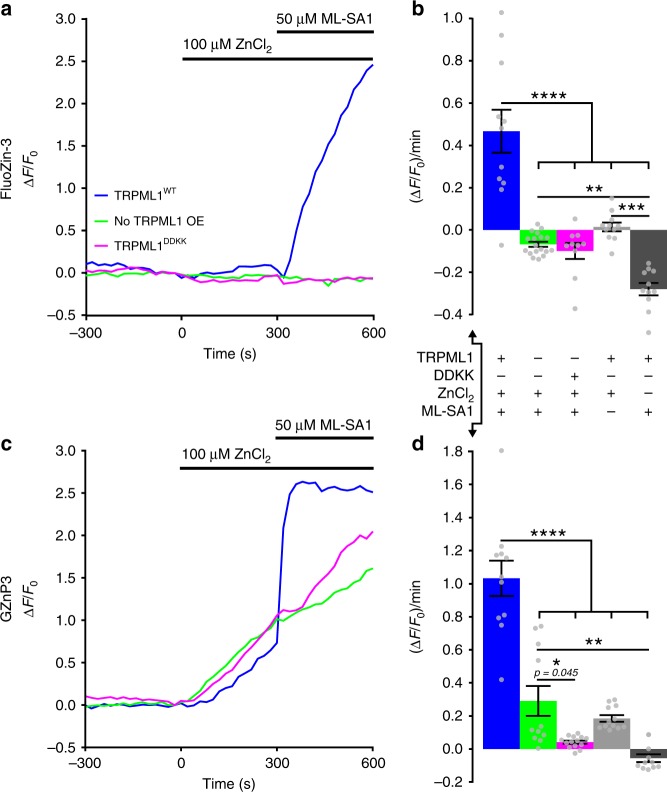
TRPML1 is permeable to physiological levels of Zn^2+^. **a** Representative traces of FluoZin-3-stained HeLa cells either un-transfected (no TRPML1 OE, green), or expressing dominant negative (TRPML1^DDKK^, magenta) or wild-type (TRPML1^WT^, blue) mCherry-TRPML1. Cells were treated with 100 µM ZnCl_2_ at 0 s then 50 µM ML-SA1 at 300 s. **b** Mean slope (±s.e.m.) of FluoZin-3 signal ((∆F/F_0_)/min) for 60 s following ML-SA1 addition for TRPML1^WT^ (blue, *n* = 11 cells), no TRPML1 OE (green, *n* = 18), TRPML1^DDKK^ (magenta, *n* = 10), TRPML1^WT^ ZnCl_2_ only (light gray, *n* = 11), TRPML1^WT^ ML-SA1 only (*n* = 11). One-way ANOVA, with post-hoc Tukey HSD. **c** Representative traces of cells expressing GZnP3 alone (no TRPML1 OE, green), or co-transfected with dominant negative (TRPML1^DDKK^, magenta) or wild-type (TRPML1^WT^, blue) mCherry-TRPML1. Cells were treated as described in **a**. **d** Mean slope (±s.e.m.) of GZnP3 signal ((∆F/F_0_)/min) for 60 s following ML-SA1 addition for TRPML1^WT^ (blue, *n* = 11 cells), no TRPML1 OE (green, *n* = 11), TRPML1^DDKK^ (magenta, *n* = 14), TRPML1^WT^ ZnCl_2_ only (light gray, *n* = 12), TRPML1^WT^ ML-SA1 only (*n* = 11). One-way ANOVA, with post-hoc Tukey HSD. ****p < 0.0001, ***p < 0.001, **p < 0.01, *p < 0.05. *p*-values displayed in italics above corresponding comparisons, where applicable. Source data are provided as a Source Data file

Patch clamp studies have recorded inward current in cells expressing constitutively active mutant TRPML1^Va^ on the plasma membrane evoked by 30 mM extracellular Zn^2+^ ^14^, suggesting that TRPML1 might be permeable to Zn^2+^. However, the physiological levels of Zn^2+^ present inside the vesicular lumen are only in the hundreds of micromolar range^30^. Given that it is difficult to characterize the channel at micromolar concentrations of Zn^2+^ via electrical current measurement, we utilized fluorescent Zn^2+^ sensors to examine the permeability of TRPML1 channel to physiological Zn^2+^ in live cells. We designed an experiment to record TRPML1-mediated Zn^2+^ influx through the plasma membrane. TRPML1 is mostly localized on lysosomes and late endosomes, but previous studies have found that a small fraction of TRPML1 can localize on the plasma membrane in cells overexpressing TRPML1^31,32^. When HeLa cells expressing TRPML1 (also referred to as TRPML1^WT^) were treated with synthetic agonist ML-SA1 (with similar potency as endogenous agonist phosphatidylinositol-3,5-bisphosphate)^33–35^ in the presence of 100 µM ZnCl_2_, we detected significantly quicker and higher Zn^2+^ influx using the small molecule sensor FluoZin-3 (Fig. 2a & b). Since FluoZin-3 resides in multiple cellular compartments, we also recorded Zn^2+^ influx into the cytosol by the GZnP3 sensor, which only resides in the cytosol and nucleus. Activation of TRPML1 accelerated cytosolic Zn^2+^ increase (***blue plot***, Fig. 2c). In control cells without TRPML1 overexpression or expressing a TRPML1 dominant negative mutant (TRPML1^DDKK^)^33^, Zn^2+^ was transported into the cytosol through a slower transport mechanism (***green and magenta plots***, Fig. 2c & d). Such slow Zn^2+^ influx was barely detected by FluoZin-3 (Fig. 2a) because GZnP3 presents higher sensitivity than FluoZin-3 in response to sub-nanomolar changes in cytosolic Zn^2+^. Compared to FluoZin-3, GZnP3 also demonstrated faster kinetics in response to TRPML1-mediated Zn^2+^ influx. In addition, control treatments with ML-SA1 alone or ZnCl_2_ alone showed no rapid increase in cytosolic Zn^2+^ (Supplementary Fig. 3a, b). These data provide evidence that TRPML1 can transport physiological levels of Zn^2+^.

### TRPML1 activation evokes cytosolic Zn^2+^ signals in neurons

**Fig. 3:**
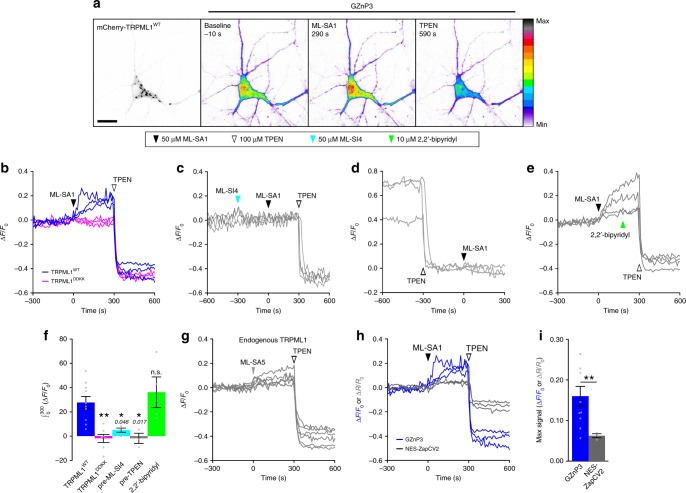
Activation of TRPML1 evokes cytosolic Zn^2+^ signals in neurons. **a** Representative micrographs of primary rat hippocampal neurons co-expressing mCherry-TRPML1^WT^ (left) and GZnP3 at baseline after ML-SA1 addition, or after TPEN treatment. Pseudocolor shows GZnP3 fluorescent intensity indicated by calibration bar (far right; minimum = white, maximum = black). Scale bar = 20 µm. **b** Representative traces of neurons co-expressing GZnP3 and mCherry-TRPML1^WT^ (blue) or mCherry-TRPML1^DDKK^ (magenta). Neurons were treated with ML-SA1 at 0 s and TPEN at 300 s. **c** Representative traces of neurons co-expressing GZnP3 and mCherry-TRPML1 pretreated with ML-SI4 at -300 s, ML-SA1 at 0 s, and TPEN at 300 s. **d** Representative traces of neurons co-expressing GZnP3 and mCherry-TRPML1 pretreated with TPEN at -300 s and ML-SA1 at 0 s. **e** Representative traces of neurons co-expressing GZnP3 and mCherry-TRPML1 treated with ML-SA1 at 0 sec, 2,2’-bipyridyl (Fe^2+^ chelator) at 180 s, and TPEN at 300 s. **f** Mean integrated GZnP3 signal (±s.e.m.) within 300 s after ML-SA1 addition for TRPML1^WT^ (blue, *n* = 10), TRPML1^DDKK^ (magenta, *n* = 7), inhibitor pretreatment (pre-ML-SI4, cyan, *n* = 4), TPEN pretreatment (pre-TPEN, gray, *n* = 3), and Fe^2+^ chelation (2,2’-bipyridyl, green, *n* = 4). One-way ANOVA with Dunnett’s multiple comparison to TRPML1^WT^. **g** Representative traces of neurons co-expressing GZnP3 and NES-mCherry treated with 10–20 µM ML-SA5 at 0 s (gray arrow) and TPEN at 300 s. **h** Representative traces of neurons co-expressing mCherry-TRPML1 and either GZnP3 (blue) or NES-ZapCV2 (gray) treated as in **b**. **i** Mean maximum sensor signal (±s.e.m.) within 300 s after ML-SA1 addition for GZnP3 (blue, *n* = 10) and NES-ZapCV2 (gray, *n* = 3). Student’s one-tailed *t*-test. **p < 0.01, *p < 0.05. n.s., not significant. *p-*values displayed in italics above corresponding comparisons where applicable. 50 µM ML-SA1 (black arrow), 100 µM TPEN (white arrow), 50 µM ML-SI4 (cyan arrow), 10 µM 2,2’-bipyridyl (green arrow). Source data are provided as a Source Data file

Next, we wanted to assess whether we could detect the release of Zn^2+^ from intracellular compartments through the TRPML1 channel. TRPML1 loss of function causes MLIV disease with a prominent neurodegenerative phenotype, so we turned our focus to a neuronal cell model. Specifically, the hippocampus has been identified as a neuronal tissue with high levels of vesicular Zn^2+^ ^36^ and hippocampal synaptic defects were observed in mouse models of MLIV^37^. As expected, in primary cultured rat hippocampal neurons co-expressing GZnP3 and mCherry-TRPML1, activation of TRPML1 with 50 µM ML-SA1 induced a significant increase in GZnP3 fluorescence, which can be quenched by the Zn^2+^ chelator, TPEN (Fig. 3a, ***blue plot*** Fig. 3b & f). No signals were detected in neurons expressing TRPML1^DDKK^ (***magenta plot*** Fig. 3b & f). Additionally, pretreatment with 50 µM ML-SI4, a TRPML1 inhibitor^38^, also blocked GZnP3 signals upon addition of ML-SA1 (Fig. 3c & f). Without overexpression of TRPML1, treatment with a more potent activator ML-SA5^38^ evoked tiny and variable GZnP3 signals through endogenous TRPML1 channels (Fig. 3g), which is similar to the weak Ca^2+^ release mediated by endogenous TRPML1^35^. In order to assess and compare the vesicular pool of Zn^2+^ that can be liberated through TRPML1 channels, we used a commonly used TRPML1 overexpression system^16,17,33,35,38,39^ that would allow rapid release of vesicular Zn^2+^ upon TRPML1 activation for all the following experiments.

We performed additional experiments to confirm that the increase in GZnP3 fluorescence was indeed due to increased Zn^2+^. Neurons pretreated with 100 µM TPEN showed no detectable increase in GZnP3 fluorescence upon addition of ML-SA1 (Fig. 3d & f). As TRPML1 is also permeable to Fe^2+^, which has also been suggested in MLIV pathology^14^, we further examined whether the GZnP3 signal was caused by a release of Fe^2+^ to the cytosol. We found that TRPML1-induced GZnP3 signals were not affected by Fe^2+^ chelator 2,2’-bipyridyl, though Zn^2+^ chelation with TPEN rapidly quenched the signal (Fig. 3e & f). As TRPML1 activation can induce proton leak from acidic lysosomes^40^, we examined whether the TRPML1-mediated Zn^2+^ signals were underestimated due to the quenching of GZnP3 by reduced pH. We simultaneously recorded the changes in pH and GZnP3 signals and corrected the effects caused by pH (see Supplementary methods). The results found that the TRPML1-mediated GZnP3 signals were only slightly lower than the pH-corrected values (Supplementary Fig. 4 & 5).

Finally, we selected ZapCV2, a previously published genetically encoded zinc sensor, to test whether it could detect a TRPML1-mediated Zn^2+^ release. Unlike GZnP3, ZapCV2 utilizes a different sensor design platform called Förster resonance energy transfer (FRET) to detect Zn^2^^+^ with nanomolar binding affinity (K_d_ = 2.3 nM). ZapCV2 yields one of the highest in situ dynamic ranges (2.2) and maximal response above baseline (0.49), but this is substantially lower than the dynamic range and maximal response above baseline of GZnP3 (11 and 4, respectively) (Table 1). In neurons co-expressing mCherry-TRPML1, NES-ZapCV2 only detected an average maximum signal increase 6.2 ± 0.5% above baseline, which was significantly smaller than the 16.0 ± 2.4% increase of GZnP3 (Fig. 3h & i). Together, these data serve as the first direct evidence of TRPML1-mediated Zn^2+^ release from intracellular stores to the cytosol in hippocampal neurons.

### Subcellular targeting of GZnP3 to detect local Zn^2+^ signals

After revealing this TRPML1-mediated Zn^2+^ release in primary rat hippocampal neurons, we sought to identify in which subcellular compartment(s) this released Zn^2+^ was stored. Though the endolysosomal localization of TRPML1 has been extensively studied in various cell types such as human skin fibroblasts^18^, HeLa^19,20^, and HEK293^14,21^, we wanted to confirm this localization in hippocampal neurons overexpressing TRPML1 and investigate if TRPML1 is localized on synaptic vesicles which contain high concentration pools of Zn^2+^.

Subcellular localization studies were carried out using primary cultured rat hippocampal neurons transfected with mCherry-TRPML1 and incorporated with markers of various subcellular compartments. TRPML1 colocalized most strongly with GFP-Rab7a, which decorates late endosomes and some lysosomes (Pearson’s correlation coefficient, PCC = 0.810 ± 0.010) (Supplementary Fig. 6a, b). TRPML1 also strongly colocalized with the acid-sensitive lysosomal dye, LysoTracker Green (Thermofisher) (PCC = 0.740 ± 0.026) (Supplementary Fig. 6a, b). However, mCherry-TRPML1 weakly localized with synaptophysin-EGFP (PCC = 0.455 ± 0.028) or FluoZin-3 (PCC = 0.561 ± 0.020) (Supplementary Fig. 6a, b). The synaptic vesicle localization was not statistically different from the negative control mitochondrial marker (mito-EGFP; PCC = 0.420 ± 0.059). These data confirm that overexpressed TRPML1 predominantly resides on endolysosomal membranes, not synaptic vesicles.

Next, we examined the distribution of Zn^2+^ among different vesicles using a small molecule Zn^2+^ sensor, FluoZin-3. Colocalization analysis of FluoZin-3 was performed against several vesicular markers in neurons. FluoZin-3 demonstrated comparable localization with both late endosomes (mCherry-Rab7a, Pearson’s R value = 0.623 ± 0.021, n = 23) and synaptic vesicles (VAMP2-RFP, Pearson’s R value = 0.678 ± 0.042, *n* = 12), suggesting that late endosomes might also make up a large subset of high-Zn^2+^ storage sites (Supplementary Fig. 6c). However, FluoZin-3 had poor colocalization with LysoTracker Red (Thermofisher) (Pearson’s R value = 0.420 ± 0.037, *n* = 8). The data suggested that Zn^2+^ is localized in both endolysosomal vesicles and synaptic vesicles, but also demonstrated non-specific localization of FluoZin-3 in various vesicles, which would limit its application to distinguish Zn^2+^ among various vesicular pools. Instead, genetically encoded sensors like GZnP3 can be specifically targeted to organelles by fusion to various targeting signal peptides or proteins. Therefore, we targeted GZnP3 onto the surface of various subcellular vesicles to distinguish local TRPML1-meditated Zn^2+^ release from different sources.

**Fig. 4:**
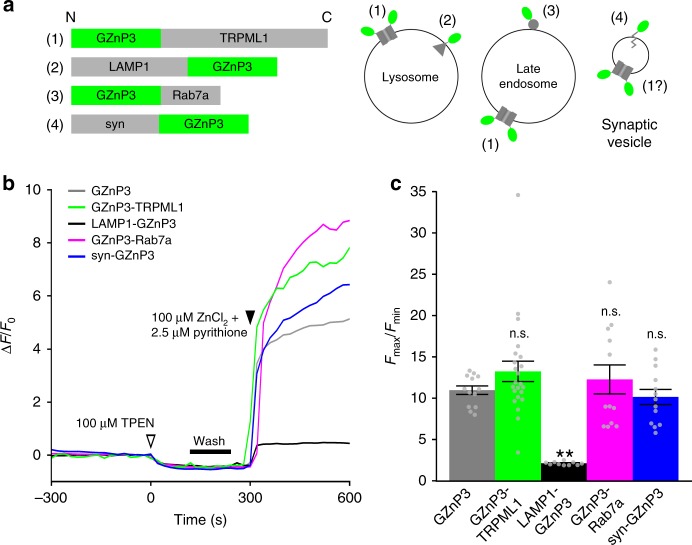
Target Zn^2+^ sensor to subcellular compartments to detect local TRPML1-mediated signals. **a** Schematic of protein constructs for (1) GZnP3-TRPML1, (2) LAMP1-GZnP3, (3) GZnP3-Rab7a, and (4) synaptophysin-GZnP3 (syn-GZnP3) with N- and C-termini marked (left). Diagram of subcellular localizations of each targeted GZnP3 construct (right). **b** Representative traces of HeLa cells expressing GZnP3 (gray), GZnP3-TRPML1 (green), LAMP1-GZnP3 (black) GZnP3-Rab7a (magenta), or synaptophysin-GZnP3 (blue). Cells were treated with 100 µM TPEN at 0 s (white arrow), washed (black bar), then treated with 100 µM ZnCl_2_ and 2.5 µM pyrithione at 300 s (black arrow). **c** Mean sensor dynamic range (±s.e.m.) of cytosolic control GZnP3 (gray, *n* = 13 cells) and vesicular-targeted variants: GZnP3-TRPML1 (green, *n* = 23), LAMP1-GZnP3 (black, *n* = 10), GZnP3-Rab7a (magenta, *n* = 12), and synaptophysin-GZnP3 (blue, *n* = 13). HeLa cells were treated as described in **b**. Bars indicate mean fold-change of sensor fluorescence (F_max_/F_min_). One-way ANOVA with Dunnett’s multiple comparison to GZnP3. ****p < 0.0001, n.s. not significant. *p*-values listed in italics above corresponding comparisons where applicable. Source data are provided as a Source Data file

To measure Zn^2+^ release directly from TRPML1 itself, we tagged GZnP3 to the cytosolic N-terminus of TRPML1 (Fig. 4a), as previously performed with Ca^2+^ sensor GCaMP3^35^. To measure lysosomal Zn^2+^ release, we fused GZnP3 to the cytosolic C-terminus of LAMP1, an integral lysosomal protein (Fig. 4a). We also attached GZnP3 to the N-terminus of Rab7a for measurement of Zn^2+^ release from late endosomes. Though our colocalizations suggested that TRPML1 has low colocalization with synaptic vesicles, we still wanted to examine if any ectopic localization of mCherry-TRPML1 on synaptic vesicles were responsible for the detected cytosolic Zn^2+^ signal. To this end, we fused GZnP3 to the cytosolic C-terminus of synaptophysin (Fig. 4a) to measure local Zn^2+^ release from synaptic vesicles. After making these new sensor constructs, we examined if the sensor response to Zn^2+^ was affected by the fusion of the marker protein because fusion with a targeting motif can potentially alter the tertiary structure of the sensor to perturb the sensor response. In HeLa cells, all vesicular-targeted sensors, except LAMP1-GZnP3, displayed dynamic ranges between approximately 10- and 13-fold, not significantly different from cytosolic GZnP3 (Fig. 4b, c). Therefore, GZnP3-TRPML1, GZnP3-Rab7a, and synaptophysin-GZnP3 could be used to sensitively detect Zn^2+^ release from various vesicular compartments.

### TRPML1 releases Zn^2+^ from endolysosomal vesicles

The targeted variants of GZnP3 were then used to measure and compare local TRPML1-mediated Zn^2+^ release from the three potential Zn^2+^ storage sites: lysosomes, late endosomes, and synaptic vesicles. In primary hippocampal neurons, GZnP3-TRPML1 had punctate vesicular morphology similar to that of mCherry-TRPML1 (Supplementary Fig. 7a). GZnP3-Rab7a showed punctate morphology that visibly colocalized with mCherry-TRPML1, though it had a slight cytosolic signal (Supplementary Fig. 7b). Matching previous data in axon terminals, synaptophysin-GZnP3 puncta were in close proximity to, but did not visibly colocalize with mCherry-TRPML1 (Supplementary Fig. 7c).

**Fig. 5:**
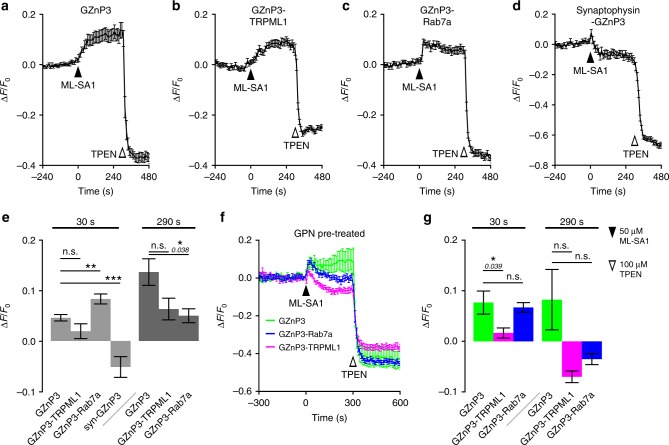
Activation of TRPML1 promotes Zn^2+^ release from lysosomes and late endosomes, not synaptic vesicles. Average traces (±s.e.m.) of primary rat hippocampal neurons **a** coexpressing mCherry-TRPML1 and GZnP3, **b** expressing GZnP3-TRPML1, **c** coexpressing mCherry-TRPML1 and GZnP3-Rab7a, or **d** coexpressing mCherry-TRPML1 and synaptophysin-GZnP3, treated with 50 µM ML-SA1 at 0 s and 100 µM TPEN at 300 s. **e** Mean normalized GZnP3 signal (±s.e.m.) 30 and 290 s after ML-SA1 addition for cytosolic GZnP3 (*n* = 10 neuron soma), GZnP3-TRPML1 (*n* = 48 puncta from 5 neuron soma), GZnP3-Rab7a (*n* = 62 puncta from nine neuron soma), and synaptophysin-GZnP3 (syn-GZnP3; *n* = 45 puncta from three neurons). Two-tailed student’s *t*-tests with Bonferroni correction for multiple comparisons. **f** Average traces (±s.e.m.) of primary rat hippocampal neurons coexpressing mCherry-TRPML1 and cytosolic GZnP3 (green), GZnP3-Rab7a (blue), or expressing GZnP3-TRPML1 (magenta), treated with 200 µM GPN from -600 to -480 seconds (not shown). **g** Mean normalized GZnP3 signal (±s.e.m.) 30 and 290 s after ML-SA1 addition for cytosolic GZnP3 (*n* = 6 neuron soma), GZnP3-TRPML1 (*n* = 30 puncta from four neuron soma), GZnP3-Rab7a (*n* = 51 puncta from six neuron soma). One-tailed student’s *t*-tests with Bonferroni correction for multiple comparisons. 50 µM ML-SA1 (black arrow), 100 µM TPEN (white arrow). ***p < 0.001, **p < 0.01, *p < 0.05, n.s. not significant. *p*-values listed in italics above corresponding comparisons where applicable. Source data are provided as a Source Data file

Activation of TRPML1 induced a gradual increase in both cytosolic GZnP3 (Fig. 5a) and GZnP3-TRPML1 fluorescence (Fig. 5b, Supplementary Fig. 8a), which were not significantly different at either 30 seconds or 290 s after TRPML1 activation with ML-SA1. Interestingly, TRPML1 activation evoked rapid GZnP3-Rab7a signals (Fig. 5c, Supplementary Fig. 8b). The GZnP3-Rab7a signal was significantly higher than cytosolic GZnP3 30 seconds after ML-SA1 addition, but was statistically lower than GZnP3 signal at 290 seconds. This suggests that GZnP3-Rab7a is able to detect local Zn^2+^ release around late endosomes before it diffuses away to the cytosol, while cytosolic GZnP3 detects cytosolic Zn^2+^ that are slowly equilibrated with Zn^2+^ release from endolysosomes. The rapid signals (<20 s) detected by GZnP3-Rab7a suggest that activation of TRPML1 channels can generate endolysosomal Zn^2+^ microdomains that are defined by localized high-Zn^2+^ concentrations. To monitor Zn^2+^ release from synaptic vesicles, we only focused on the axons and axon terminals that contain large amounts of synaptic vesicles^41^ to avoid picking up signals from the endosomes (synaptophysin has slight endogenous localization on endosomes^42^ and overexpression might enhance non-synaptic localization). Though synaptic vesicles colocalized strongly with FluoZin-3, treatment with ML-SA1 induced no significant synaptophysin-GZnP3 signal (Fig. 5d, Supplementary Fig. 8c), which suggests that the majority of TRPML1-mediated Zn^2+^ signals are not released from synaptic vesicles.

To further distinguish late endosomes from lysosomes, we pretreated cells with 200 µM glycyl-L-phenylalanine 2-naphthylamide (GPN) for two minutes to disrupt lysosomes by osmotic lysis^43^. After GPN pretreatment, both GZnP3 and GZnP3-Rab7a detected TRPML1-mediated Zn^2+^ release (Fig. 5f, g, Supplementary Fig. 9a, c). However, GZnP3-TRPML1 had little to no signal (Fig. 5f, g, Supplementary Fig. 9b), showing similar response as synaptophysin-GZnP3 trace without GPN treatment (Fig. 5d). The GZnP3-Rab7a and GZnP3-TRPML1 signal intensities both slowly decayed over ~5 min, however, possibly due to dilution of Zn^2+^ throughout the cytosol, Zn^2+^ reuptake into vesicles, or membrane injury and clearance caused by lysosomal disruption. Together, our data suggest that GZnP3-Rab7a can monitor Zn^2+^ release from late endosomes and TRPML1 activation can release Zn^2+^ from both lysosomes and late endosomes.

### Comparison of TRPML1-mediated Zn^2+^ and Ca^2+^ in neurons

**Fig. 6:**
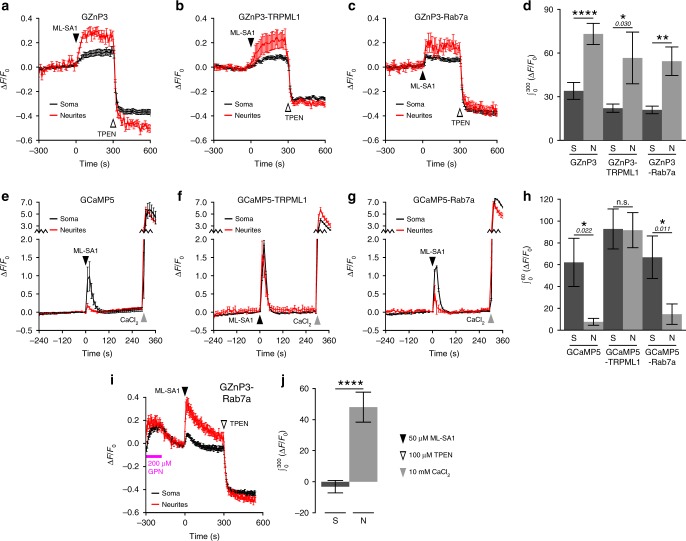
TRPML1 mediates greater Zn^2+^ release and less Ca^2+^ release in neurites than in the soma. Average traces (±s.e.m.) from soma (black) or neurites (red) of primary rat hippocampal neurons co-expressing **a** mCherry-TRPML1 and GZnP3, **b** expressing GZnP3-TRPML1, or **c** co-expressing mCherry-TRPML1 and GZnP3-Rab7a. **d** Mean integrated GZnP3 signal (±s.e.m.) 300 s after ML-SA1 addition between soma (dark) and neurites (light) for GZnP3 (soma, *n* = 10; neurites, *n* = 70 puncta), GZnP3-TRPML1 (soma, *n* = 48 puncta; neurites, *n* = 54 puncta), and GZnP3-Rab7a (soma, *n* = 62 puncta; neurites, *n* = 28 puncta). One-tailed Student’s *t*-tests. Average traces (±s.e.m.) from soma (black) or neurites (red) of primary rat hippocampal neurons **e** co-expressing mCherry-TRPML1 and GCaMP5, **f** expressing GCaMP5-TRPML1, or **g** co-expressing GCaMP5-Rab7a and mCherry-TRPML1. **h** Mean integrated GCaMP5 signal (±s.e.m.) 60 s after ML-SA1 addition between soma (dark) and neurites (light) for GCaMP5 (soma, *n* = 8; neurites, *n* = 68 puncta), GCaMP5-TRPML1 (soma, *n* = 30 puncta; neurites, *n* = 66 puncta), and GCaMP5-Rab7a (soma, *n* = 25 puncta; neurites, *n* = 42 puncta). One-tailed Student’s *t*-tests. **i** Average traces (±s.e.m.) from soma (black) and neurites (red) of primary rat hippocampal neurons co-expressing GZnP3-Rab7a and mCherry-TRPML1 pretreated with 200 µM GPN (magenta bar). **j** Mean integrated GZnP3-Rab7a signal (±s.e.m.) 300 s after ML-SA1 addition to neurons pretreated with GPN between soma (dark) and neurites (light) (soma, n = 41 puncta; neurites, n = 59 puncta). 50 µM ML-SA1 (black arrow), 100 µM TPEN (white arrow), 10 mM CaCl_2_ (gray arrow). One-tailed Student’s *t*-test. ****p < 0.0001, **p < 0.01, *p < 0.05, n.s. not significant. *p*-values listed in italics above corresponding comparisons where applicable. S, soma. N, neurites. Source data are provided as a Source Data file

Neurons are highly polarized with functionally different structures including the soma, axons, and dendrites. Recent work discovered that endolysosomal vesicles are not evenly distributed among the soma and neural processes. Rab7-positive late endosomes are found throughout the dendrites, while the mature and degradative lysosomes are mostly concentrated in the soma^44^. For this reason, we compared TRPML1-mediated Zn^2+^ signal intensity between the soma and neurites. Interestingly, there were significantly higher TRPML1-mediated Zn^2+^ signals in neurites than the soma detected by cytosolic GZnP3 (Fig. 6a, d), GZnP3-Rab7a (Fig. 6c, d), and GZnP3-TRPML1 (Fig. 6b, d).

In order to examine the possibility that the higher Zn^2+^ signals in neurites are due to the spatially limited diffusion of Zn^2+^ ions along these thin processes or different distributions of endolysosomal vesicles, we compared TRPML1-mediated Ca^2+^ signals between the soma and neurites by using GCaMP5, a sensitive green fluorescent calcium sensor extensively used in neuronal calcium studies^45^. Similar to GZnP3, GCaMP5 was fused to the N-terminus of both Rab7a and TRPML1 to measure Ca^2+^ release from late endosomes and lysosomes. Cytosolic GCaMP5 showed a rapid spike in fluorescence upon addition of ML-SA1 and then immediate decay (Fig. 6e–g), consistent with previously reported TRPML1-mediated Ca^2+^ signals in retinal pigment epithelium^46^, COS-1^39^, CHO^35^, and HeLa^39^ cells. Different from Zn^2+^ signals, the TRPML1-mediated Ca^2+^ signals were significantly lower in neurites as compared to the soma as detected by GCaMP5 (Fig. 6e, h) and GCaMP5-Rab7a (Fig. 6g, h). Conversely, GCaMP5-TRPML1 showed no significant difference between soma and neurites (Fig. 6f, h). Such data suggest that the endolysosomes, especially late endosomes store higher levels of Zn^2+^, and less Ca^2+^ in neurites than in the soma.

To further verify that the higher Zn^2+^ signals in neurites were due to more Zn^2+^ released from the Rab7a-positive late endosomes in neurites than the soma, we compared the TRPML1-mediated Zn^2+^ signal between neurites and soma after lysosomal Zn^2+^ was depleted in neurites with 200 µM GPN. GZnP3 showed slightly higher signals in neurites than the soma, while GZnP3-TRPML1 had no observable signal in either soma or neurites after lysosomal disruption (Supplementary Fig. 10a, b). As expected, after GPN treatment, GZnP3-Rab7a detects significantly higher Zn^2+^ release from late endosomes in neurites than the soma (Fig. 6i, j), indicating that Zn^2+^ in the Rab7a-positive late endosomes are higher in neurites than in the soma. Overall, our data suggest that the Rab7a-positive late endosomes store higher Zn^2+^ and less Ca^2+^ in neurites than the soma.

Interestingly, it was also observed that unlike the Ca^2+^ signals, the Zn^2+^ signals did not return quickly to baseline after ML-SA1 treatment. Such difference was not caused by variations in sensor kinetics because GZnP3 showed comparable or even faster turn-on and turn-off responses compared with GCaMP5 (Supplementary Fig. 11, see Supplementary Methods). Sustained Zn^2+^ signals might be due to slower buffering mechanisms than Ca^2+^. To test this, we utilized a depolarization condition to accumulate high Ca^2+^ and Zn^2+^ in the cytosol^47^. After depolarization was removed, Ca^2+^ was reduced by 99%, while Zn^2+^ was only buffered 12% within 15 min (Supplementary Fig. 12, see Supplementary Methods). Therefore, the cytosolic Zn^2+^ signals, either released from vesicular compartments or induced by influx, can last longer than Ca^2+^.

### Cell heterogeneity of TRPML1-mediated Zn^2+^ and Ca^2+^ signals

**Fig. 7:**
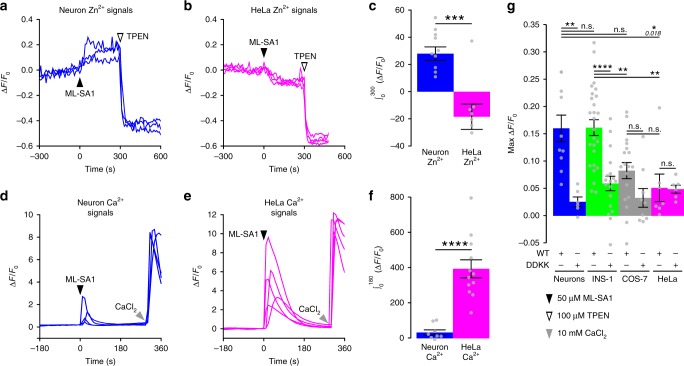
TRPML1-mediated Zn^2+^ signals are higher in neurons and INS-1 cells than other mammalian cell types. **a** Representative traces of primary rat hippocampal neurons co-expressing GZnP3 and mCherry-TRPML1. Neurons were treated with 50 µM ML-SA1 at 0 s (black arrow) and 100 µM TPEN at 300 s (white arrow). **b** Representative traces of HeLa cells co-expressing GZnP3 and mCherry-TRPML1. Cells were treated as described in **a**. **c** Mean integrated GZnP3 signal (±s.e.m.) 300 s after ML-SA1 addition for both neurons (blue, *n* = 10 cells) and HeLa cells (magenta, *n* = 7). Two-tailed Student’s *t*-test. **d** Representative traces of primary rat hippocampal neurons co-expressing GCaMP5 and mCherry-TRPML1. Neurons were treated with 50 µM ML-SA1 at 0 s (black arrow) and 10 mM CaCl_2_ at 300 s (gray arrow). **e** Representative traces of HeLa cells co-expressing GCaMP5 and mCherry-TRPML1. Cells were treated as described in **d**. **f** Mean integrated GCaMP5 signal (±s.e.m.) 180 s after ML-SA1 addition for both neurons (blue, *n* = 8) and HeLa cells (magenta, *n* = 12). Two-tailed Student’s *t*-test. **g** Mean GZnP3 signal maximum (±s.e.m.) 300 s after ML-SA1 addition for both TRPML1^WT^ and TRPML1^DDKK^ in neurons (blue; WT, *n* = 10; DDKK, *n* = 7), INS-1 cells (green; WT, *n* = 26; DDKK, *n* = 18), COS-7 cells (gray; WT, *n* = 18; DDKK, *n* = 9), and HeLa cells (magenta; WT, *n* = 7; DDKK, *n* = 6). One-way ANOVA with post-hoc Tukey HSD. ****p < 0.0001, ***p < 0.001, **p < 0.01, *p < 0.05, n.s. not significant. *p*-values listed in italics above corresponding comparisons where applicable. Source data are provided as a Source Data file

TRPML1 has nearly ubiquitous tissue expression^48^, though TRPML1 loss of function in MLIV patients presents a strong neurodegenerative phenotype. Therefore, we compared TRPML1-mediated Zn^2+^ signals among different cell types. In contrast to neurons (Fig. 7a), HeLa cells co-expressing TRPML1-mCherry and GZnP3 showed a decrease in GZnP3 signal upon activation of TRPML1 (Fig. 7b, c), likely due to decreased pH. HeLa cells showed a statistically lower Zn^2+^ release than neurons (Fig. 7c), indicating the endolysosomal vesicles may not store high Zn^2+^ in HeLa cells. We also sought to analyze whether TRPML1-mediated Ca^2+^ release was different in HeLa cells as compared to neurons. HeLa cells co-expressing GCaMP5 and mCherry-TRPML1^WT^ were treated with 50 µM ML-SA1. HeLa cells showed much larger Ca^2+^ release upon TRPML1 activation, which decayed much slower than in neurons (Fig. 7e). Unlike Zn^2+^ signals, the TRPML1-mediated Ca^2+^ signals were significantly higher in HeLa cells than in neurons (Fig. 7d–f).

Next, we sought to investigate whether the TRPML1-mediated Zn^2+^ release varied across other commonly used mammalian cell models such as the pancreatic beta-cell line (INS-1) and African green monkey kidney fibroblast-like cell line (COS-7). Interestingly, there was no significant difference between TRPML1-mediated Zn^2+^ signals in INS-1 as compared to neurons (Fig. 7g). Similar to neurons, INS-1 cells expressing mCherry-TRPML1 also showed a significantly larger TRPML1-mediated Zn^2+^ signal than INS-1 cells expressing TRPML1^DDKK^ (Fig. 7g). Similar to HeLa cells, Zn^2+^ release was not significantly higher in COS-7 cells expressing mCherry-TRPML1^WT^ as compared to TRPML1^DDKK^ (Fig. 7g). The data suggest that the neurons and pancreatic beta cells contain high Zn^2+^ in the endolysosomal vesicles that can be liberated through the TRPML1 channel, while HeLa cells and COS-7 cells lack such endolysosomal Zn^2+^ pools.

## Discussion

Though Zn^2+^ has been unequivocally identified as a vital metal ion for cellular health, the dynamics of labile Zn^2+^ have not been well characterized due to its low intracellular concentration and a lack of sensitive probes that are able to detect its physiological fluctuations in the sub-nanomolar to nanomolar range. While a growing toolbox of fluorescent Ca^2+^ sensors have been established, which vary in their sensitivity and fluorescent spectra, there are limited tools available to study Zn^2+^ signaling dynamics in live cells.

The development of GZnP3 has filled such a gap for the scientific community giving an unprecedented ability to study cellular Zn^2+^ dynamics with sub-nanomolar sensitivity in real time. GZnP3 binds labile Zn^2+^ with a K_d_ of 1.3 nM, giving it the ability to detect this metal ion in the sub-nanomolar range. Though other genetically encoded Zn^2+^ probes have similar binding affinities (Table 1), GZnP3 has approximately an 11-fold dynamic range from its apo state to Zn^2+^ saturation (17-fold in vitro), making it the most sensitive protein-based Zn^2+^ sensor currently available for monitoring sub-nanomolar cellular Zn^2+^ dynamics, between 100 pM and 1 nM. It has high specificity for Zn^2+^ over a range of other biologically relevant cations, including Ca^2+^ and Fe^2+^. Together, these characteristics permit the use of GZnP3 to observe minute changes in cytosolic [Zn^2+^] from the high picomolar to low nanomolar range. Though it is a powerful tool, it does have limitations for its use in the dynamic cellular environment. Primarily, like many GFP-based fluorophores, we are aware of its sensitivity to changes in pH. To account for this, however, we have established a normalization method to obtain pH-corrected GZnP3 signals by simultaneously recording GZnP3 (Zn^2+^) and pHuji^49^ (pH) signals (Supplementary Fig. 5).

Here, we provided the first direct evidence that Zn^2+^ can be released from intracellular compartments to the cytosol and showed that TRPML1 channels can mediate such Zn^2+^ release in neurons. One of the major challenges of current biological studies of cellular Zn^2+^ signals is to distinguish Zn^2+^ among various vesicular compartments. In this work, we created sensors that allow us to record and compare Zn^2+^ release from lysosomes, late endosomes, and synaptic vesicles. These constructs revealed several important findings. Our results support the presence of localized Zn^2+^ signals in microdomains near endolysosomes before equilibrium is reached. Additionally, GZnP3-TRPML1 and GZnP3-Rab7a can detect different pools of Zn^2+^ release. GZnP3-TRPML1 tends to detect Zn^2+^ release from lysosomes because it failed to detect Zn^2+^ signals when lysosomes were disrupted by GPN. Instead, GZnP3-Rab7a can still detect Zn^2+^ release from non-lysosomal vesicles after lysosomal disruption (Fig. 5f, g). Although high pools of Zn^2+^ are present in the synaptic vesicles^50^ (Supplementary Fig. 6c), synaptophysin-GZnP3 close to the presynaptic vesicles at axon terminals failed to detect significant Zn^2+^ signals when TRPML1 channels are activated (Fig. 5d, e). In addition, TRPML1 channel has weak colocalization with synaptic vesicles (Supplementary Fig. 6a, b). Overall, we conclude that the majority of TRPML1-mediated Zn^2+^ release is from endolysosomal compartments.

Interestingly, we found that TRPML1-mediated Zn^2+^ signals are especially higher in neurites than the soma and the majority of such higher Zn^2+^ was released from Rab7a-positive late endosomes. Biochemical evidence revealed that Zn^2+^ can inhibit cathepsin (lysosomal protease) activity at concentrations as low as 100 nM^51^, thus our findings integrate well into recent work about the polarized distributions of endolysosomal vesicles and degradative activity in neurons:^52^ mature lysosomes with high degradative activity are localized within the soma of neurons, while non-degradative Rab7a-positive late endosomes are distributed along the dendrites^44^. The negative correlation between endolysosomal Zn^2+^ and lysosomal degradative function in the neuronal soma and neurites implicates the potential involvement of Zn^2+^ in lysosomal degradative activity and MLIV pathology.

Additionally, there are stark differences between the signal of TRPML1-mediated Zn^2+^ release versus TRPML1-mediated Ca^2+^. First, TRPML1-mediated Ca^2+^ release in neurons shows a rapid spike and almost immediate decay, while TRPML1-mediated Zn^2+^ release is relatively slower than Ca^2+^ and has prolonged elevation. Such differences might be caused by different metal ion buffering mechanisms. There are several cellular mechanisms that regulate Ca^2+^ reuptake into various compartments including the mitochondrial Ca^2+^ uniporter (MCU) and ER Ca^2+^ ATPase (SERCA), or export from the cell through Plasma Membrane Ca^2+^ ATPase (PMCA)^53^, ensuring the fast recovery of Ca^2+^ back to baseline concentrations. In nearly all cell types, Ca^2+^ has multiple downstream signaling targets, and these sequestration mechanisms can prevent cytotoxicity from prolonged elevation of ectopic cytosolic Ca^2+^^54^. In contrast, Zn^2+^ is buffered by relatively slower mechanisms including Zn^2+^ buffering proteins^55,56^, such as metallothionein and Zn^2+^ export transporters (ZnT family)^6,8^. Such slow mechanisms might allow the small amount of released Zn^2+^ signals to effectively interact with Zn^2+^ sensing proteins such as metal transcription factor MTF1^57^, tyrosine phosphatase^58^, and other signaling molecules to regulate neuron function. Second, there is an inverse correlation between TRPML1-mediated Zn^2+^ and Ca^2+^. TRPML1-mediated Zn^2+^ signals are higher in neurons than HeLa cells, while TRPML1-mediated Ca^2+^ signals are lower in neurons than HeLa cells (Fig. 7). In addition, when TRPML1 is activated in neurons, Zn^2+^ signals are higher in neurites than soma, while Ca^2+^ signals are lower in neurites than soma. This negative correlation suggests that increase in Zn^2+^ in the endolysosomal vesicles might be complemented with a decrease in Ca^2+^. The increase of Zn^2+^/Ca^2+^ ratio in the endolysosomal vesicles in neurons, especially in the neurites, might be associated with unique roles of these vesicles in neuron function.

In summary, we reported the creation of new Zn^2+^ probes GZnP3, GZnP3-TRPML1, and GZnP3-Rab7a for detection of sub-nanomolar Zn^2+^ dynamics. With these tools, we provided the first direct evidence that TRPML1 can mediate Zn^2+^ release from endolysosomal vesicles to the cytosol. Such Zn^2+^ signals demonstrate cell specificity and are especially higher in neurites than the soma in neurons. Overall, our work provides instrumental tools to investigate the biological function of endolysosomal Zn^2+^ pools and implicates TRPML1-mediated Zn^2+^ signals in proper neuronal function.

## Methods

### Animals

Pregnant Sprague Dawley rats were purchased from Charles River (strain: 400). Animal treatment and maintenance were performed by the University of Denver Animal Facility (AAALAC accredited). All experimental procedures using animals were approved by the Institutional Animal Care and Use Committee (IACUC) of the University of Denver.

### In vitro characterization of K_d_, metal specificity

For sensor protein expression, the sensor was cloned into the pBAD vector and expressed in Top10 *Escherichia coli* upon addition of 0.2% arabinose. Sensor protein was purified by Ni^2+^ ion affinity chromatography using Ni-NTA chelating sepharose beads, and the 6X Histidine tag was removed with a homemade TEV protease treated in 20 mM Tris, 100 mM NaCl, and a pH 8 buffer at RT overnight (sensor to TEV protease ratio was 10:1). The sensor protein was stored at 4 ˚C in the dark for up to one week from the day of purification. Zn^2+^ titrations were performed in HEPES buffer (150 mM HEPES, 100 mM NaCl, 0.5 mM TCEP and 10% glycerol, pH 7.4) with 2 μM sensor protein and Zn^2+^ buffering solutions with defined Zn^2+^ concentrations^5^. The sensor was reduced with TCEP for 10 min prior to performing analysis. Purified sensor protein was titrated with Zn^2+^ to determine the fluorescence intensity as a function of Zn^2+^ concentration. The sensor response to Zn^2+^ was reported as an apparent dissociation constant (K_d_’), which reflects the Zn^2+^ concentration midway between the F_min_ and F_max_. Metal selectivity was measured using 2 µM purified sensor protein in HEPES buffer^29^. The sensors were treated with 12 µM EDTA followed by 50 µM of each metal (ZnCl_2_, CaCl_2_, MgCl_2_, CuCl_2_, MnCl_2_, CoCl_2_, NiSO_4_, FeCl_2_, KCl, FeCl_3_, AlCl_3_, CrCl_3_). FeCl_2_ was maintained in the reduced oxidation state (Fe^2+^) using ascorbic acid as a reducing agent to prevent oxidation to Fe^3+^^59^. The in vitro fluorescence measurements used for Zn^2+^ titrations and metal specificity studies were made on a Tecan fluorescence plate reader using the following parameters: excitation, 488 nm; emission, 515 nm; emission bandwidth, 5 nm.

### Biophysical characterization of GZnP3

All biophysical characterizations were performed on purified sensor in 30 mM MOPS (pH 7.4), 100 mM KCl, and 0.5 mM TCEP. The quantum yield was determined using fluorescein as a reference, diluted in 0.1 M NaOH^29^, over a dilution series between 0.01 to 0.05 AU absorbance at 494 nm using a Varian Cary 100 Bio UV-Vis spectrophotometer. Sensor proteins were incubated with 10 µM ZnCl_2_ or 15 µM TPA (Zn^2+^ chelator) to obtain the quantum yield of the sensor in the bound and apo state, respectively. The same dilution series was then run through a Cary Eclipse fluorescence spectrophotometer fluorimeter with excitation at 488 nm and emission from 500 nm to 600 nm using 1 nm slits, 1 second integration time, and 1 nm steps. The total fluorescence intensities were obtained by integrating the emission spectrum of each sample (500–600 nm) and plotted against the absorbance reading at the maximum wavelength (494 nm). The quantum yield was obtained from the slopes of each line and by plugging into the equation Φ_Sensor_ = Φ_Standard_ × (Slope of sensor/Slope of standard); Φ_Standard_ = 0.925 for fluorescein. Extinction coefficients were calculated from the absorbance spectrum for a set concentration of purified sensor in the presence of 15 µM TPA and 10 µM ZnCl_2_. The sensor protein concentrations were determined using A446 nm after alkali denaturation with 0.1 M NaOH for 3 min to eliminate fluorescence and generate an absorbance for Ɛ_446 nm_. The extinction coefficient was calculated using the equation Ɛ_sensor_/44,000 = Absorbance of sensor/Absorbance in NaOH.

### DNA constructs

Plasmids were constructed by molecular cloning and verified by sequencing. Site directed mutagenesis was used to introduce mutations to designed positions on GZnP1^29^. GZnP3 was fused to Rab7a amplified from mCherry-Rab7a (Addgene #55127) and TRPML1 from TRPML1-HA (Addgene #18825)^21^. GZnP3 was fused to LAMP1 amplified from LAMP1-RFP (Addgene #1817)^60^. GZnP3 was fused to synaptophysin from synaptophysin-GCaMP3 (a kind gift from Dr. Susan M Voglmaier, UCSF). GZnP1, GZnP2, GZnP3, and pHuji (amplified from TfR-pHuji, Addgene #61505)^49^ were amplified and cloned into the pDisplay vector.

### Non-neuronal cell culture and transfection

HeLa cells were maintained in high glucose Dulbecco’s Modified Eagle Medium (DMEM) with 10% fetal bovine serum (FBS) at 37 °C, 5% CO_2_. INS-1 cells were maintained in Roswell Park Memorial Institute (RPMI) 1640 Medium supplemented with 10 mM HEPES, 5 mM sodium pyruvate, 50 µM 2-mercaptoethanol, and 10% FBS. PEI (polyethyleneimine) transfection reagent was used for the transfection in non-neuronal cells (dissolved to 1 mg/ml in water at pH 7.2 with long-term storage at −20 ˚C, short term storage at 4 °C). Transfection was performed when the cells were ~40–50% confluent. For each transfection reaction in non-neuronal cells, 2–3 µL of PEI transfection reagent and 1–1.25 µg of DNA were mixed in 250 µL Opti-MEM. The mixture was incubated for a minimum of 25 min at room temperature before direct addition to one imaging dish containing the cells.

### Primary rat hippocampal neuron culture and transfection

Primary hippocampal neurons were prepared from rat embryos at embryonic day 18 (E18) in dissociation medium containing 10X HBSS, 1 M HEPES buffer (pH 7.3) and 50 µg/mL gentamycin. The hippocampi were minced and treated with 1000 U/mL papain added to the dissection medium, and dissociated by trituration in 1 mg/mL DNase I. Cells were plated on 1 mg/mL poly-L-lysine-coated 14 mm round glass coverslips at a density of 40,000 cells/coverslip per dish in neuron plating medium (MEM supplemented with glucose and 5% FBS). After checking that cells were adhered, neuron plating medium was replaced with Neurobasal medium (Thermofisher) supplemented with 0.3X GlutaMAX (Thermofisher) and 1X B-27 (Thermofisher). Cultures were maintained at 37 °C, 5% CO_2_. Neurons were transfected between DIV 6-15, using the Lipofectamine 3000 transfection kit (Thermofisher) in 500 µL Opti-MEM. The reagent-DNA mixture was incubated for a minimum of 25 min at room temperature before direct addition to the neuron imaging dishes. Before adding reagent-DNA, 1 mL media was removed from each imaging dish and syringe filtered with an equal volume of fresh neuron culture media (50:50 media). After incubation at 37 °C for 4 h, neurons were washed three times with 1 mL prewarmed neuron culture media. Then, 2 mL of the 50:50 media was added to the neuron imaging dishes and they were incubated at 37 °C until imaging.

### Live cell microscopy

Cells were imaged 48 h post-transfection, and cells were washed three times with the indicated imaging buffer immediately before imaging. All imaging was performed on an inverted Nikon/Solamere CSUX1 spinning disc confocal microscope with a ×40 (for time-lapse) or ×60 (for colocalization) 1.4 NA oil immersion objective. Data were collected using MicroManager software and analyzed with Fiji (ImageJ).

### Colocalization

Colocalizations with mCherry-TRPML1: Between DIV 6-15, primary cultured rat hippocampal neurons were either transfected only with mCherry-TRPML1, or co-transfected with a late endosomal marker (GFP-Rab7a) or a synaptic vesicle marker (synaptophysin-EGFP). After 48 h, neurons that were only transfected with mCherry-TRPML1 were loaded with either 1 µM LysoTracker Green (Thermofisher) or 2 µM FluoZin-3 (Thermofisher). To determine the c.

Colocalizations with FluoZin-3: Between DIV 6-15, primary cultured rat hippocampal neurons were either transfected with mCherry-TRPML1^WT^, mCherry-TRPML1^DDKK^, mCherry-Rab7a, or VAMP2-RFP. After 48 h, neurons were loaded with 2 µM FluoZin-3, and neurons that were un-transfected were simultaneously loaded with 1 µM LysoTracker Red (Thermofisher).

All neurons were then washed and imaged in Zn^2+^-free buffer. Colocalizations were performed using the Coloc 2 plugin for ImageJ. Point spread functions for the ×60 objective were measured for both 488/525 nm (full width at half maximum, FWHM = 2.58 pixels, PSF 1st σ = 154.5 nm) and 561/605 nm (FWHM = 2.75 pixels, PSF 1st σ = 164.9 nm) excitation/emission wavelengths and an average FWHM of 2.663 pixels was used as the input for the Coloc 2 plugin. To determine the Pearson’s coefficient for random localization or autocorrelation, the colocalization was analyzed between TRPML1-mcherry and mito-EGFP that have no biological association as a negative control.

### In situ metal specificity

For in situ Ca^2+^ specificity, HeLa cells were transfected with GZnP3 and imaged in phosphate-free HHBSS (containing 1.26 mM Ca^2+^). After collecting a 5-min baseline, 10 µM ionomycin (in DMSO) was added to rapidly influx Ca^2+^ into the cytosol. After 5 min, 100 µM TPEN was added to the cells to chelate all Zn^2+^. For in situ Fe^2+^ specificity, HeLa cells were transfected with GZnP3 and imaged in 0 Ca^2+^ phosphate-free HHBSS. After collecting a 5-min baseline, 10 µM 2,2’-bipyridyl (in DMSO) was added to chelate cellular Fe^2+^. After 5 min, 100 µM TPEN was added to the cells to chelate all Zn^2+^.

### pDisplay sensor sensitivity assay

HeLa cells were pretreated with 100 µM TPEN to bring sensor fluorescence to its minimum, then cells were washed and imaged with buffer containing 500 µM TCEP to prevent sensor oxidation. Starting at the proposed resting cytosolic concentration, we tested the sensors’ response across a thousand-fold range of physiologically relevant concentrations: 100 pM, 1 nM, 10 nM, and 100 nM. Standard solutions of various Zn^2+^ concentrations (Zn^2+^/Chelator-buffered solutions) were prepared at defined concentrations^5^. Images were acquired every 10 s, with a 200 ms exposure of 488 nm laser excitation at 10 mW power. A stable baseline was collected for 120 s before the addition of Zn^2+^.

### TRPML1 activation in neurons

Primary cultured rat hippocampal neurons were transfected between 6 and 15 days in vitro (DIV). After 48 h, neurons were washed and imaged in 0 Ca^2+^, 0 Zn^2+^ HHBSS. After collecting a baseline signal for 5 min, neurons were treated with 50 µM ML-SA1 (or 10–20 µM ML-SA5 where indicated) to open TRPML1 channels. For inhibition experiments, after baseline was collected, 50 µM ML-SI4 was added for five minutes before ML-SA1 addition. As an internal control to confirm the presence of functional sensors, 100 µM TPEN was added to quench intracellular Zn^2+^ for cells expressing GZnP3, or 10 mM CaCl_2_ was added to influx Ca^2+^ for cells expressing GCaMP5. For GZnP3 and GCaMP5 variants, images were acquired every 5–10 seconds, with a 200 ms exposure of 488 nm laser excitation at 10 mW power. For ZapCV2, images were acquired every 10 seconds with 200 ms exposures of 445 nm laser excitation at 10 mW power, capturing emissions at both 480 nm (CFP) and 535 nm (FRET). The background corrected FRET and CFP intensities were used to calculate the FRET ratio (R).

### Testing Dynamic Range of vesicular-targeted GZnP3 variants

HeLa cells were imaged in 0 Zn^2+^, phosphate-free HHBSS buffer, 48 h post-transfection. After a 5 min baseline, cells were treated with 100 µM TPEN to collect the minimum sensor fluorescence (F_min_). TPEN was then removed by washing the cells three times with 0 Zn^2+^ buffer. Finally, 100 µM ZnCl_2_ and 2.5 µM pyrithione (a Zn^2+^ ionophore) were added simultaneously to influx Zn^2+^ and generate maximum sensor fluorescence (F_max_). F_max_ was then divided by F_min_, representing the sensor’s dynamic range of intensity as a fold-change relative to its minimum fluorescence. Images were acquired every 20 s, with a 200 ms exposure of 488 nm laser excitation at 10 mW power.

### Data Analysis

Imaging data were analyzed with Fiji (ImageJ) and raw data output from Fiji were analyzed using Excel in combination with JMP software (JMP^®^, Version 13.0. SAS Institute Inc., Cary, NC). Statistical analysis was performed using Excel or JMP software, either performing unpaired *t*-tests or one-way ANOVA with appropriate post-hoc corrections. All *t*-tests were preceded by an F-test to analyze variance equality. *t*-tests were then performed with consideration for the determined variance equality or inequality. ANOVA with comparisons to a single control used Dunnett’s post-hoc correction, and ANOVA with comparisons across all groups used post-hoc Tukey HSD. All measurements were taken from distinct samples, though multiple regions of interest (ROIs) were selected from a single neuron for measuring distinct puncta or various unique structures. No ROI was measured repeatedly. When selecting a ROI in the soma for cytosolic sensors that were also present in the nucleus, care was taken to avoid selecting nuclear areas. For cells loaded with FluoZin-3, care was taken to avoid high intensity puncta likely representing vesicles with high-Zn^2+^ or concentrated dye. For time traces, background fluorescence was subtracted from ROIs. For single wavelength sensors, changes in fluorescent intensity (∆F = F−F_0_) were normalized to the baseline preceding the 0 second time point (F_0_), indicated as ∆F/F_0_. For FRET sensors, images were acquired for both the acceptor and donor emission wavelengths, and the changes in the ratio of acceptor to donor emission was calculated for each time point (∆R = R−R_0_). Again, these signals were normalized to the baseline preceding the 0 s time point (R_0_), indicated as ∆R/R_0_.

### Reporting summary

Further information on research design is available in the Nature Research Reporting Summary linked to this article.

## Supplementary information

Supplementary information

Transparent Peer Review File

Reporting Summary

Source Data

## Data Availability

Original data are available from the corresponding author upon reasonable request. Source data underlying Figs. 1–7 and Supplementary Figures 1-6, 10-12 are provided as a Source Data file.
