# Supplementary material for: Sub-nanomolar sensitive GZnP3 reveals TRPML1-mediated neuronal Zn2+ signals
Source: Nat Commun. 2019 Oct 22;10:4806. doi: 10.1038/s41467-019-12761-x (PMC6805855; doi:10.1038/s41467-019-12761-x)
Supplement: Supplementary file 1 — Supplementary information [file 41467_2019_12761_MOESM1_ESM.pdf]

Sub-nanomolar sensitive GZnP3 reveals TRPML1-mediated neuronal Zn^{2+} signals

Minckley et al., 2019

Supplementary Methods

***in situ* pH normalization of GZnP3 signal**

To normalize GZnP3 signal to correct for Zn^{2+}-independent, pH-dependent changes, we calculated the changes in sensor intensity across a physiologically relevant range of pH (5 to 9) in HeLa cells. Using the pDisplay assay with either GZnP3 or the red fluorescent protein-based pH sensor, pHuji, we performed *in situ* pH normalization. Buffers were made at pH 5.1, 5.5, 6, 6.5, 7, 7.4, 8, 8.5 and 8.9, using phosphate free HHBSS imaging buffer with identical components except the pH buffers changed depending on the desired pH (MES for pH 5.1, 5.5, and 6; MOPS for pH 6.5; HEPES for pH 7, 7.4, and 8; Tris for pH 8.5 and 8.9). pDisplay-pHuji and pDisplay-GZnP3 were transfected into cells and imaged 48 hours post-transfection. Images were acquired every 20 seconds, with 100 ms exposure at 10 mW laser power for the 488 nm laser and 50 ms and 5 mW for the 561 nm laser. Cells were imaged for 5 minutes in each pH buffer, then washed with the next sequential pH buffer, beginning with pH 5.1 and ending at pH 8.9. Averages of each pH's sensor intensity for both the GZnP3 and pHuji sensor were taken to plot intensity of the sensor as a function of pH. These intensities were normalized to physiological pH (7.4). The normalized values for each sensor were plotted against each other to allow the estimated change in GZnP3 intensity as a function of change in pHuji intensity.

Using the pDisplay assay, we generated standard curves for each fluorescent protein normalized to fluorescence at physiological pH 7.4 (**Supplementary Fig. 4a & 4b**). We then plotted corresponding GZnP3 values against pHuji values to generate a standard curve (**Supplementary Fig. 4c**) which we could use to normalize GZnP3 signal by intracellular pH changes as measured by the pHuji sensor (**Supplementary Equations 3 and 4**).

We then simultaneously measured pH and TRPML1-mediated Zn^{2+} release in neurons co-expressing GZnP3-TRPML1 and pHuji. This allowed us to observe proton leak through TRPML1 at the same time as normalizing the GZnP3 signal to account for the pH change. As expected, neurons showed a slight cytosolic acidification upon TRPML1 activation (**magenta line, Supplementary Fig. 5a**). We normalized the GZnP3-TRPML1 signal (**green line, Supplementary Fig. 5a**) to remove pH-dependent changes. As lower pH was shown to reduce GZnP3 signal (**Supplementary Fig. 4c**), this normalization showed that the intensity of the detected TRPML1-mediated Zn^{2+} signal was slightly underestimated, but not significant (**blue line, Supplementary Fig. 5a**).

Supplementary Equation 1: ***GZnP3 intensity (y) as a function of pH (x).***

$$y = 0.54322 + \frac{-1.4458}{1 + \left(\frac{x}{7.2184}\right)^{20.347}}$$

Supplementary Equation 2: ***pHuji intensity (y) as a function of pH (x).***

$$y = 11.201 + \frac{-12.13061}{1 + \left(\frac{x}{10.409}\right)^{7.6328}}$$

Supplementary Equation 3: ***GZnP3 intensity (y) as a function of pHuji intensity (x).***

$$y = 0.65638(1 - e^{-1.0696x})$$

Supplementary Equation 4: pH Normalized ***GZnP3 intensity (y) as a function of GZnP3 (z) and pHuji intensity (x).***

$$y = z - 0.65638(1 - e^{-1.0696x})$$

***in situ* kinetics analysis of GZnP2, GZnP3 and GCaMP5**.

To compare the *in situ* kinetics of GZnP2, GZnP3, or GCaMP5, HeLa cells expressing each sensor were treated with their respective cation and ionophore respectively to measure the turn-on response. Then the turn-off responses were measured for GZnP3 and GCaMP5 when ions were chelated by metal chelators or cleared away by intracellular mechanisms.

For GZnP2 and GZnP3 kinetics analysis, HeLa cells were transfected with GZnP2 or GZnP3 and imaged in 0 Ca^{2+}, 0 Zn^{2+} phosphate-free HHBSS. After collecting a 2-minute baseline, 10 µM $ZnCl_2$ and 2.5 µM pyrithione (in DMSO) was added to rapidly load Zn^{2+} into the cytosol. After 3 minutes, Zn^{2+} and pyrithione were washed away and the cells were allowed to incubate in 0 Ca^{2+}, 0 Zn^{2+}, phosphate-free HHBSS. 6 minutes after the initial Zn^{2+} influx, 100 µM TPEN was added to the cells to chelate all Zn^{2+}. For GZnP3, linear regression analysis was performed for each trace corresponding to a single cell, for both Zn^{2+} influx and efflux. For GZnP2, each trace was analyzed for the time to reach 95% sensor saturation.

For GCaMP5 kinetics analysis, HeLa cells were transfected with GCaMP5 and imaged in 0 Ca^{2+}, 0 Zn^{2+} phosphate-free HHBSS. After collecting a 2-minute baseline, 1 mM $CaCl_2$ and 5 µM ionomycin (in DMSO) was added to induce Ca^{2+} influx into the cytosol. After 3 minutes, Ca^{2+} and ionomycin were washed away and the cells were allowed to incubate in 0 Ca^{2+}, phosphate-free HHBSS. 6 minutes after the initial Ca^{2+} influx, 5 mM EGTA and 5 µM ionomycin was added to the cells to chelate all Ca^{2+}. Linear regression analysis was performed for each trace corresponding to a single cell, for both Ca^{2+} influx and efflux. Since Ca^{2+} had already returned to baseline by the time EGTA was added, and EGTA didn't reduce intensity below baseline, efflux measurements were taken from the rapid decrease upon washout of extracellular Ca^{2+} and ionomycin.

For GZnP2, GZnP3, and GCaMP5, images were acquired every 2.5 seconds, with a 200 ms exposure of 488 nm laser excitation at 10 mW power.

Zn^{2+} and Ca^{2+} recovery after depolarization.

In order to compare the buffering rate of Ca^{2+} vs Zn^{2+}, we loaded neurons with 1 mM Ca^{2+} or 100 µM Zn^{2+} under similar conditions. Neurons were depolarized with 50 mM KCl, whereby voltage-dependent Ca^{2+} channels (VGCCs) opened to allow Ca^{2+} and Zn^{2+} entry.

Primary cultured rat hippocampal neurons were transfected with GZnP3 or GCaMP5 at 12 days *in vitro* (DIV). After 48 hours, neurons were washed and imaged in 0 Ca^{2+}, 0 Zn^{2+} HHBSS. After collecting a baseline signal for 5 minutes, neurons were treated with 100 µM $ZnCl_2$ (for GZnP3) or 1 mM $CaCl_2$ (for GCaMP5) and 50 mM KCl to open VGCCs by depolarization. After 5 minutes, neurons were washed with 0 Ca^{2+}, 0 Zn^{2+} HHBSS, and neurons were incubated for 15 minutes. Images were acquired every 10 seconds, with a 200 ms exposure of 488 nm laser excitation at 10 mW power.

Supplementary Table 1: ***In vitro biophysical characterization of GZnP3 sensor.*** Listed below are the apparent dissociation constant (K_d), Hill coefficient (*n*), and the Quantum yields (QY) and extinction coefficients for both the Zn^{2+} bound and unbound states of GZnP3.

Apparent dissociation constant (K_d, pH 7.4)	Hill coefficient (*n*, pH 7.4)	Quantum Yield (QY, Apo)	Quantum Yield (QY, Zn^{2+}-bound)	Extinction coefficient (Apo)	Extinction coefficient (Zn^{2+}-bound)
1.3 nM	0.34	0.112	0.462	4,000	23,400

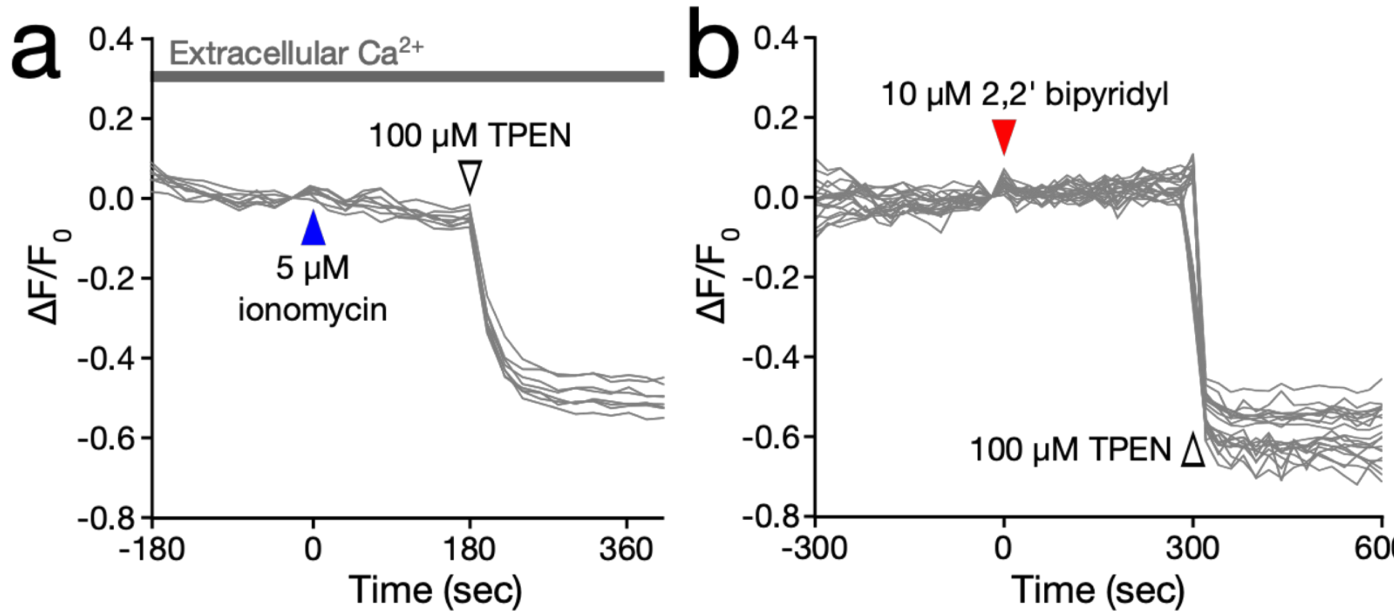

Supplementary Figure 1: ***In situ metal specificity of GZnP3.***
(a) *In situ* metal specificity for Ca^{2+} in HeLa cells expressing cytosolic GZnP3. Cells were imaged in media with 1.26 mM Ca^{2+}, and treated with 5 µM ionomycin at 0 sec (blue arrow) and 100 µM TPEN at 300 sec (white arrow). Each trace represents a cell, n = 8. (**b**) *In situ* metal specificity for Fe^{2+} in HeLa cells expressing cytosolic GZnP3. Cells were imaged in Ca^{2+} free media, and treated with 10 µM 2,2'-bipyridyl at 0 sec (red arrow) and 100 µM TPEN at 300±10 sec (white arrow). Each trace represents a cell, n = 21. Source data are provided as a Source Data file.

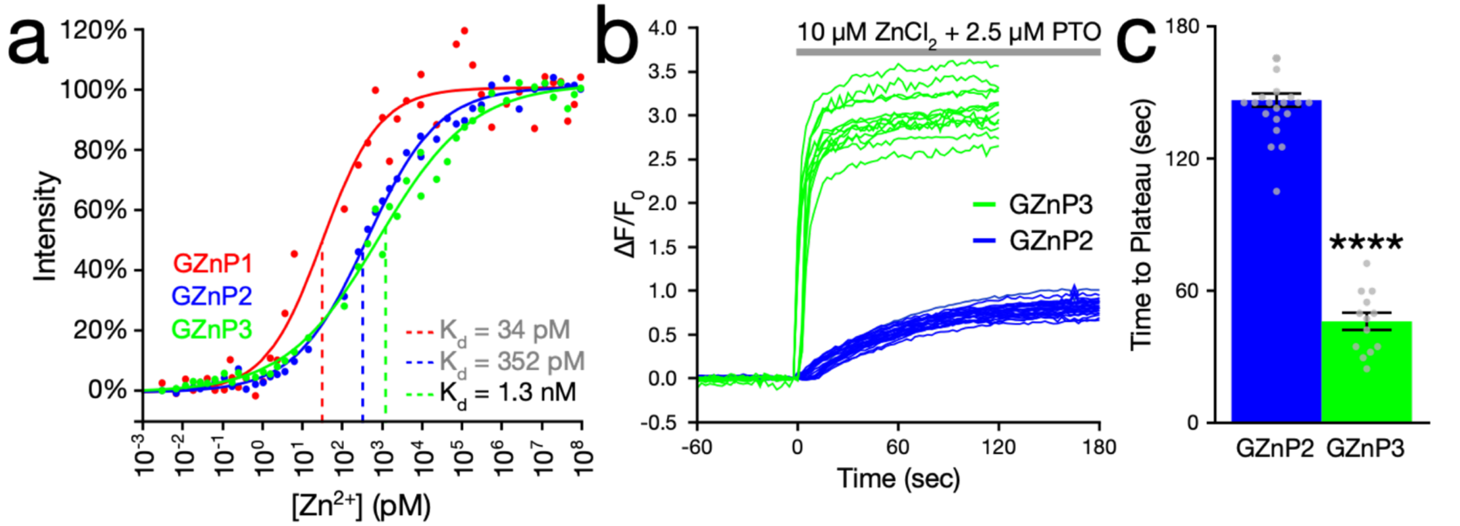

Supplementary Figure 2: ***Compare binding affinity and kinetics of GZnP family sensors.***
(a) *In vitro* Zn^{2+} binding curves for GZnP1 (red), GZnP2 (blue), and GZnP3 (green) are shown at pH 7.4. Sigmoidal binding curves were fitted for each sensor. Intensity data is normalized to each respective binding curve. Vertical dashed lines show respective dissociation constants for GZnP1 (K_d = 34 pM), GZnP2 (K_d = 352 pM), and GZnP3 (K_d = 1.3 nM). **(b)** Representative traces of HeLa cells expressing GZnP3 (green) or GZnP2 (blue), treated with 10 µM $ZnCl_2$ and 2.5 µM pyrithione (PTO) at 0 seconds. **(c)** Mean time (±SEM) for each sensor in (b) to reach signal plateau for GZnP3 (green, n = 13 cells) and GZnP2 (blue, n = 24 cells) in (b). One-tailed Student's t test. **** $p < 0.0001$. Source data are provided as a Source Data file.

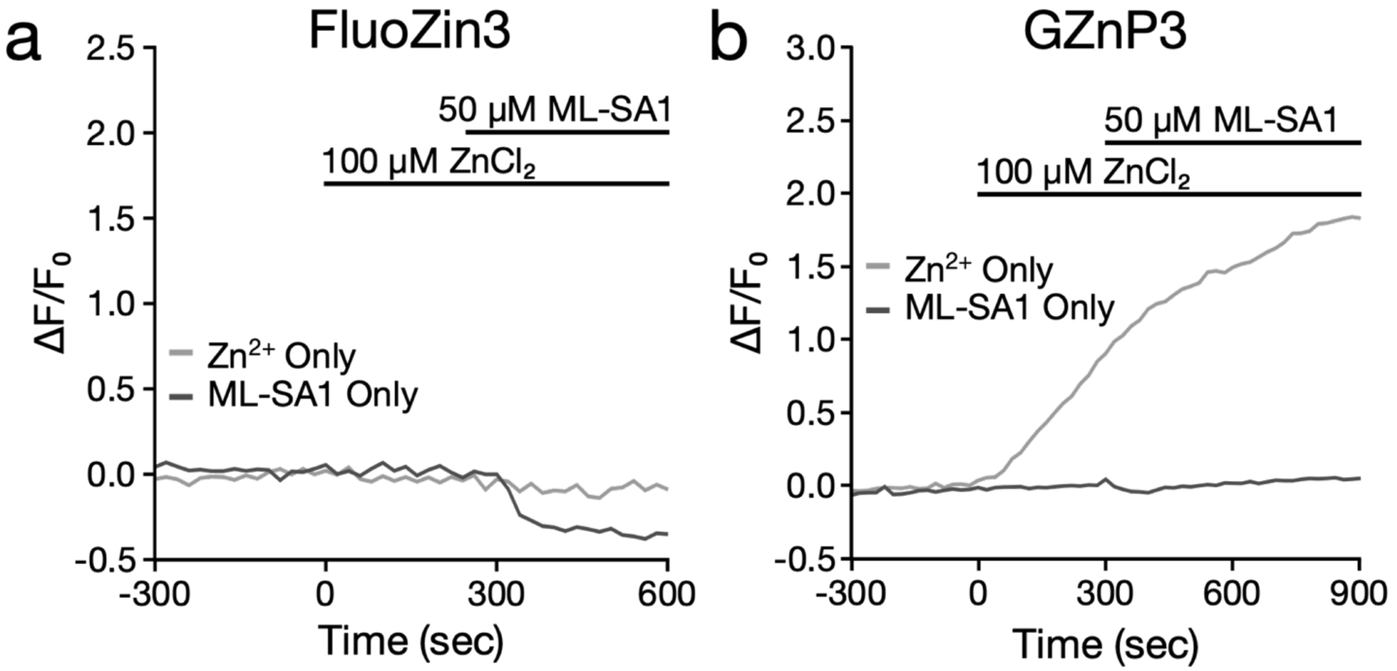

Supplementary Figure 3: ***Compare FluoZin-3 and GZnP3 signals in cells treated with $ZnCl_2$ or ML-SA1 alone.***
(a) Representative traces of FluoZin-3-stained cells overexpressing mCherry-TRPML1, treated with either only 100 µM $ZnCl_2$ at 0 sec or 50 µM ML-SA1 at 300 sec. Quantification included in Figure 2b. **(b)** Representative traces of cells expressing GZnP3 and mCherry-TRPML1, treated with either only 100 µM $ZnCl_2$ at 0 sec or 50 µM ML-SA1 at 300 sec. Quantification included in Figure 2d. Source data are provided as a Source Data file.

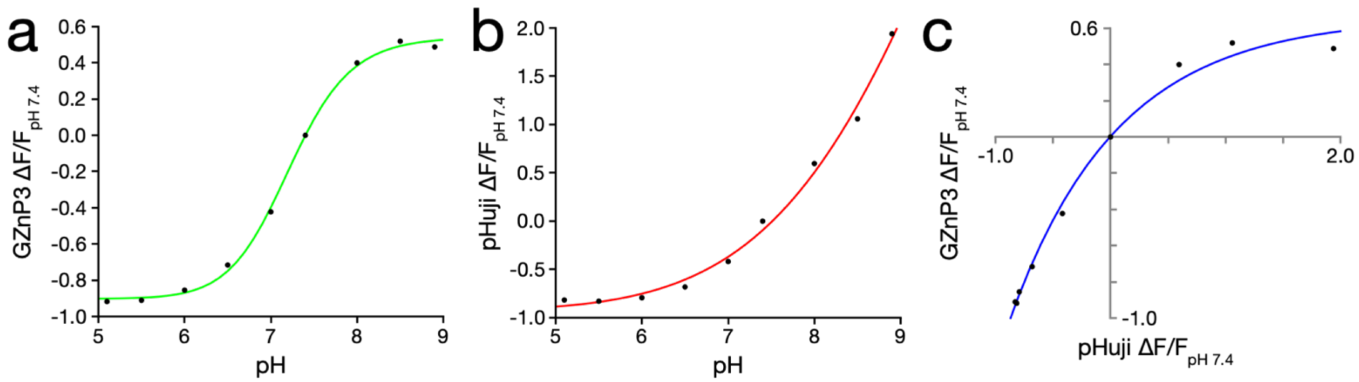

Supplementary Figure 4: ***pH effect and pHuji normalization of GZnP3 sensor.***
(a) The change in fluorescent intensity of pDisplay-GZnP3 was plotted against pH. The sensor intensity was normalized to physiological pH (7.4). Green line shows fitted sigmoidal curve, R = 0.99927, see Supplementary Equation 1. **(b)** The change in fluorescent intensity of pDisplay-pHuji was plotted against pH. The sensor intensity was normalized to physiological pH (7.4). Red line shows fitted exponential curve, R = 0.99658, see Supplementary Equation 2. **(c)** pH effect on GZnP3 intensity. The y-axis values from the graphs in (a) and (b) were plotted against each other to display the change in GZnP3 signal as a function of detected change in the pHuji signal. Blue line shows fitted exponential rise curve, R = 0.99596, see Supplementary Equation 3. This allows for direct normalization of intracellular GZnP3 signal by removing pH-dependent changes in brightness detected by pHuji, see Supplementary Equation 4. Source data are provided as a Source Data file.

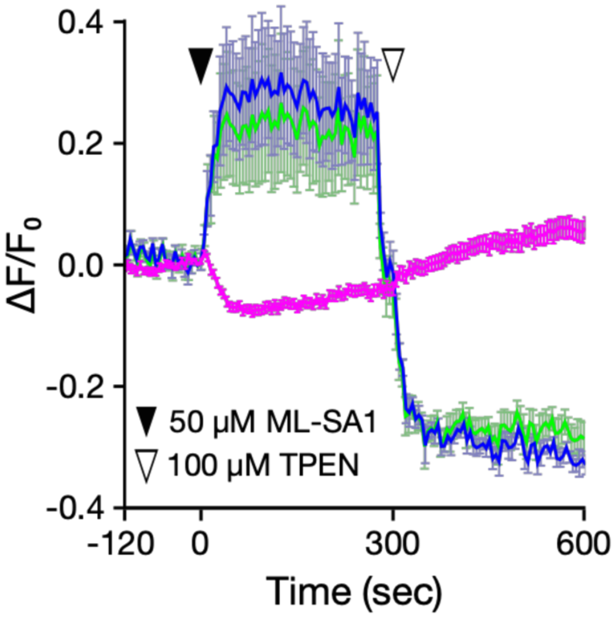

Supplementary Figure 5: ***pH corrected signal shows ML-SA1 induced signal detects Zn^{2+} independent of pH.***
Average traces (± SEM) of primary rat hippocampal neurons co-expressing GZnP3-TRPML1 (green) and pHuji (magenta). GZnP3 fluorescence was normalized to pH using Supplementary Equation 4. Neurons were treated with 50 µM ML-SA1 at 0 sec (black arrow) and 100 µM TPEN at 300 sec (white arrow). 3 neurons, n = 33 puncta. Source data are provided as a Source Data file.

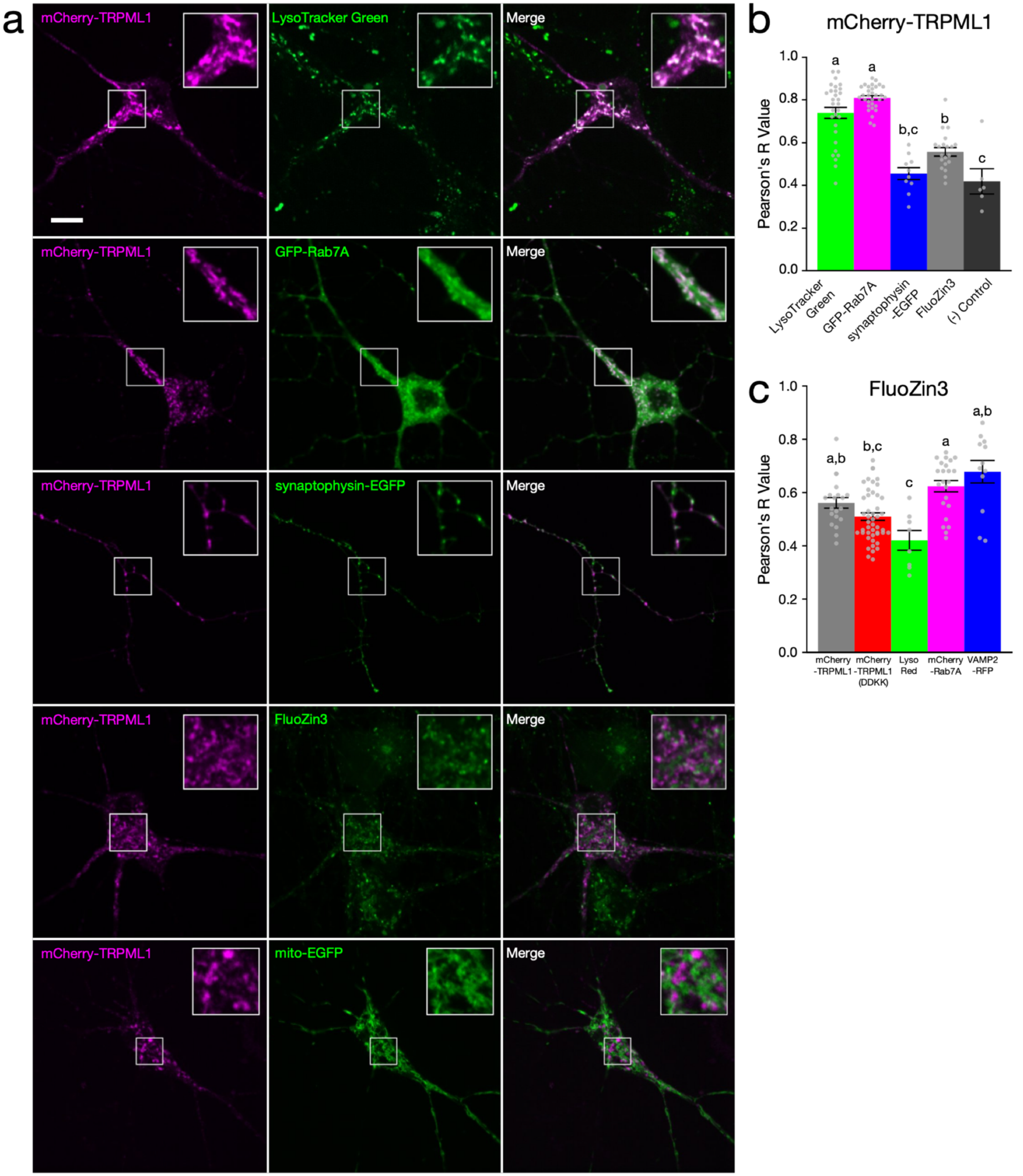

Supplementary Figure 6: ***Determine subcellular localization of overexpressed TRPML1 and FluoZin-3 puncta.***
(a) Representative confocal micrographs of primary cultured rat hippocampal neurons expressing mCherry-TRPML1 and either loaded with 1 μM LysoTracker Green (lysosomal marker, top) or 2 μM FluoZin-3 (Zn^{2+} sensing dye, row 4), or co-transfected with GFP-Rab7a (late endosomal marker, row 2), synaptophysin-EGFP (synaptic vesicle marker, row 3), or negative control mito-EGFP (mitochondrial marker, bottom). Images were acquired following excitation at 561 nm (magenta, left) or 488 nm (green, middle), and the channels were merged to show colocalization (right). The inset at the top

right of each micrograph shows detail (2.5x enlarged) of vesicular structures (white box). Scale bar = 10 µm. **(b)** Mean Pearson's R values (± SEM) for mCherry-TRPML1 with Lysotracker Green (green, n = 30 neurons), GFP-Rab7a (magenta, n = 31 neurons), synaptophysin-EGFP (blue, n = 10 neurons), FluoZin-3 (light gray, n = 20 neurons), or negative control mito-EGFP (dark gray, n = 6 neurons). One-way ANOVA, with post-hoc Tukey HSD. Levels not connected by same letter are significantly different, $p < 0.0001$ for all except FluoZin-3 compared to negative control ($p = 0.0397$). **(c)** Mean Pearson's R Value (±SEM) for cells loaded with 2 µM FluoZin-3, overexpressing mCherry-TRPML1^{WT} (gray, n = 20), mCherry-TRPML1^{DDKK} (red, n = 46), mCherry-Rab7a (magenta, n = 23), VAMP2-RFP (blue, n = 12) or cells simultaneously loaded with 1 µM LysoTracker Red (green, n = 8). One-way ANOVA, with post-hoc Tukey HSD. Levels not connected by same letter are significantly different, $p < 0.01$ for all except TRPML1 compared to LysoTracker ($p = 0.0195$). Source data are provided as a Source Data file.

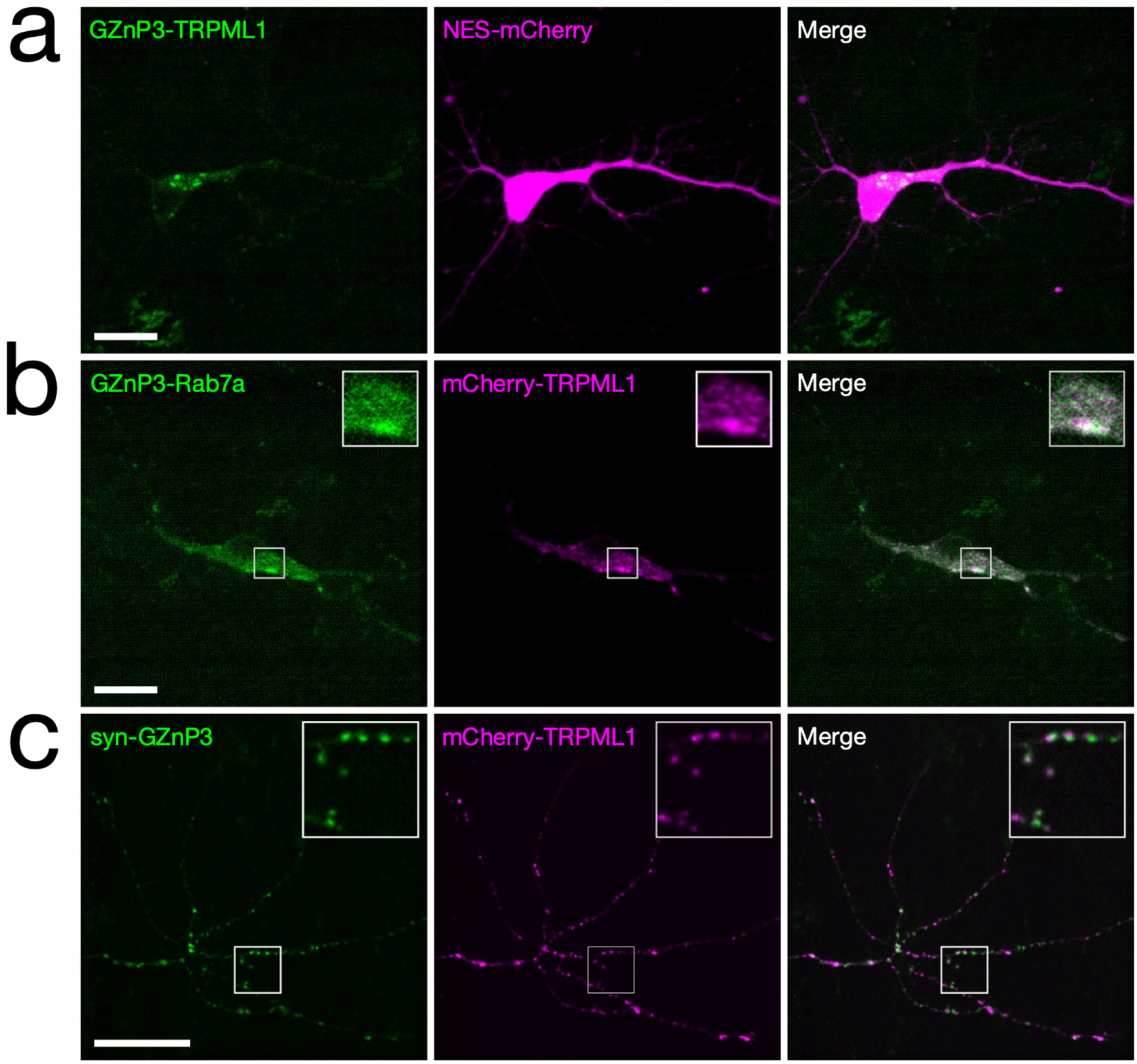

Supplementary Figure 7: ***Vesicular-tagged GZnP3 constructs in primary hippocampal neurons.***
Representative confocal micrographs of primary cultured rat hippocampal neurons coexpressing **(a)** NES-mCherry and GZnP3-TRPML1, or mCherry-TRPML1 and either **(b)** GZnP3-Rab7a or **(c)** synaptophysin-GZnP3 (syn-GZnP3). Images were acquired following excitation at 488 nm (green, left) or 561 nm (magenta, center), and the channels were merged to

show localization (right). The inset at the top right shows detail (2.5x enlarged) of selected areas (white box). Scale bars = 20 µm.

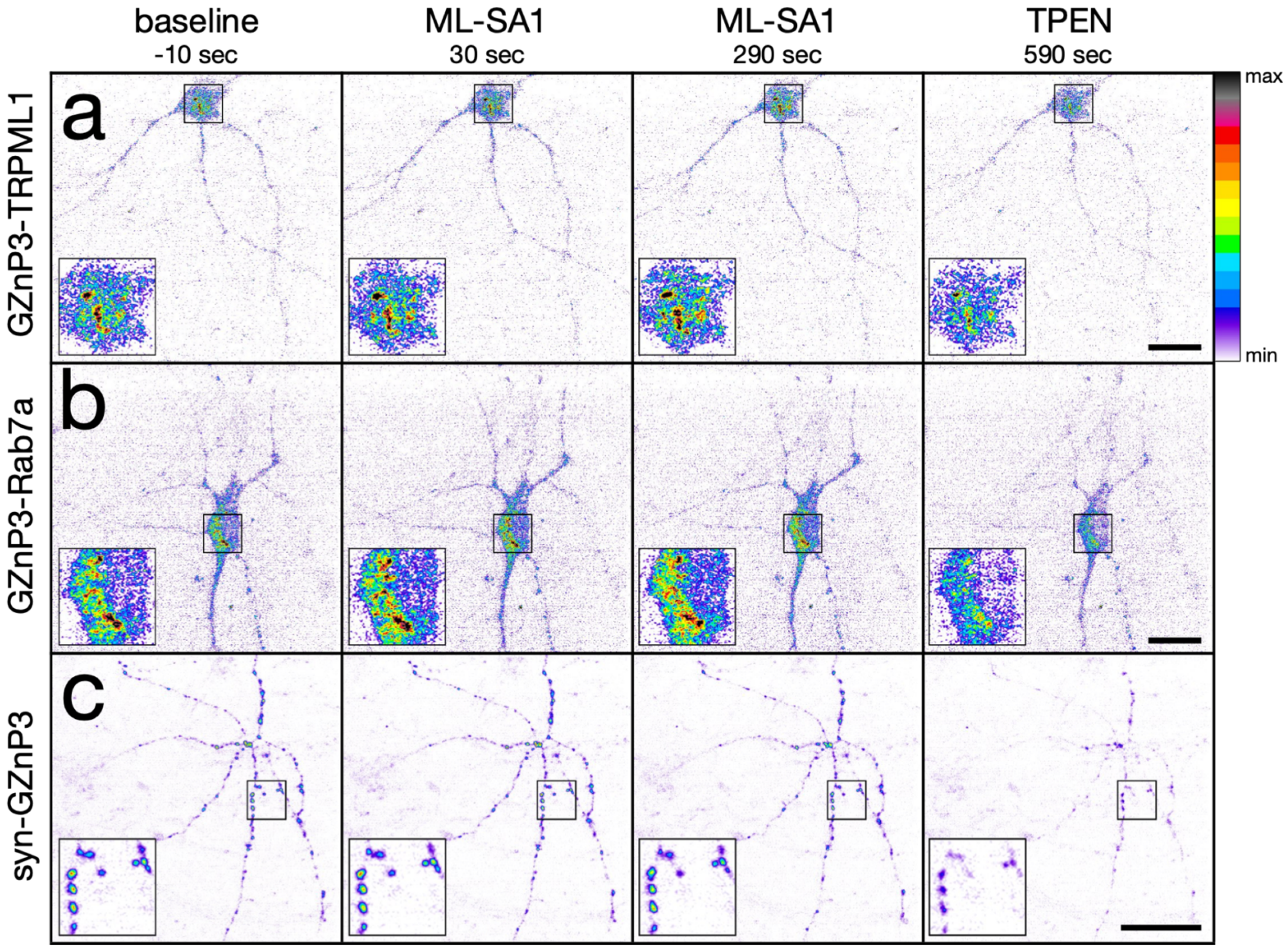

Supplementary Figure 8: ***GZnP3 sensor constructs response to TRPML1-activation.***
Representative confocal micrographs of primary cultured rat hippocampal neurons expressing **(a)** GZnP3-TRPML1, or co-expressing mCherry-TRPML1 and either **(b)** GZnP3-Rab7a or **(c)** synaptophysin-GZnP3 (syn-GZnP3). From left to right: sensors at baseline, 30 seconds after 50 µM ML-SA1 addition, 290 seconds after ML-SA1 addition, and after 100 µM TPEN treatment. Pseudocolor shows GZnP3 fluorescent intensity indicated by calibration bar (far right; minimum = white, maximum = black). The inset at the bottom left shows detail (2.5x enlarged) of selected areas (black box). Scale bars = 20 µm.

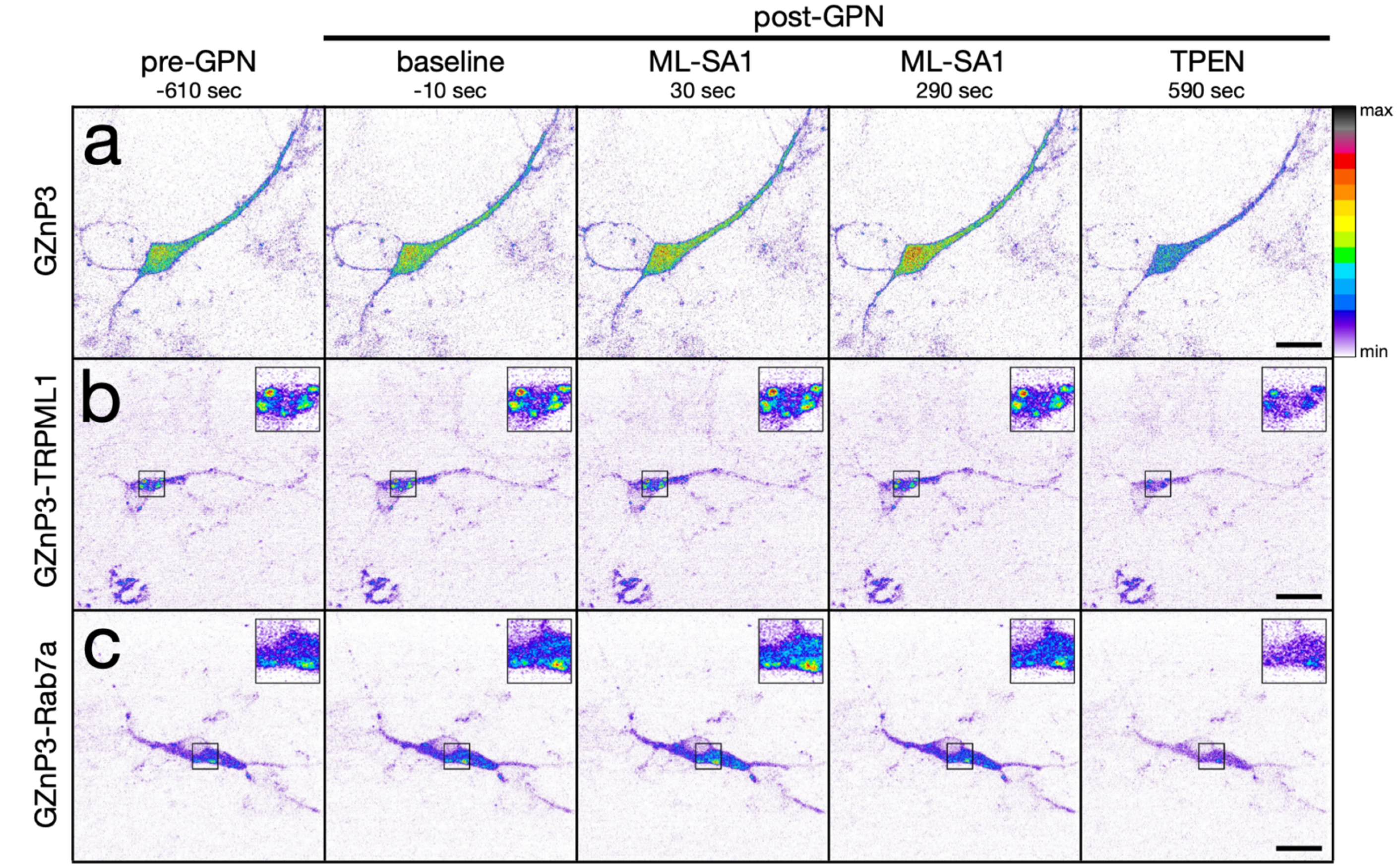

Supplementary Figure 9: ***GZnP3 sensor constructs response to TRPML1-activation after GPN pretreatment.*** Representative confocal micrographs of primary cultured rat hippocampal neurons **(a)** co-expressing mCherry-TRPML1 and GZnP3, **(b)** expressing GZnP3-TRPML1, or **(c)** co-expressing mCherry-TRPML1 and GZnP3-Rab7a. From left to right: sensors before 200 µM GPN treatment at -600 seconds, post-GPN baseline, 30 seconds after 50 µM ML-SA1 addition, 290 seconds after ML-SA1 addition, and after 100 µM TPEN treatment. Pseudocolor shows GZnP3 fluorescent intensity indicated by calibration bar (far right; minimum = white, maximum = black). The inset at the top right shows detail (2.5x enlarged) of selected areas (black box). Scale bars = 20 µm.

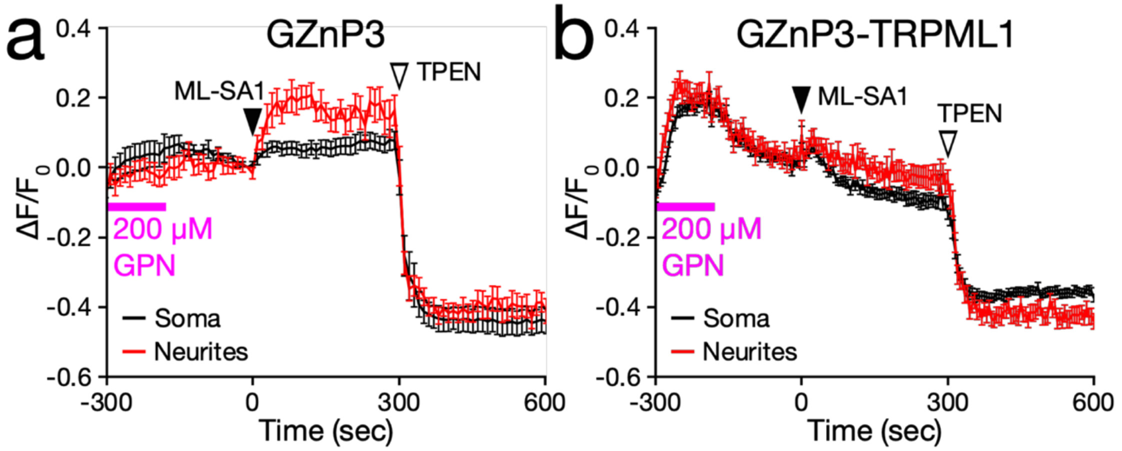

Supplementary Figure 10: ***TRPML1-mediated Zn^{2+} is not released from lysosomes in neurites.***
Average traces (± SEM) from soma (black) and neurites (red) of primary rat hippocampal neurons **(a)** co-expressing GZnP3 and mCherry-TRPML1 or **(b)** only expressing GZnP3-TRPML1, pretreated with 200 µM GPN at -300 to -180 sec (magenta

bar). Neurons were treated with 50 µM ML-SA1 at 0 sec (black arrow) and 100 µM TPEN at 300 sec (white arrow). Source data are provided as a Source Data file.

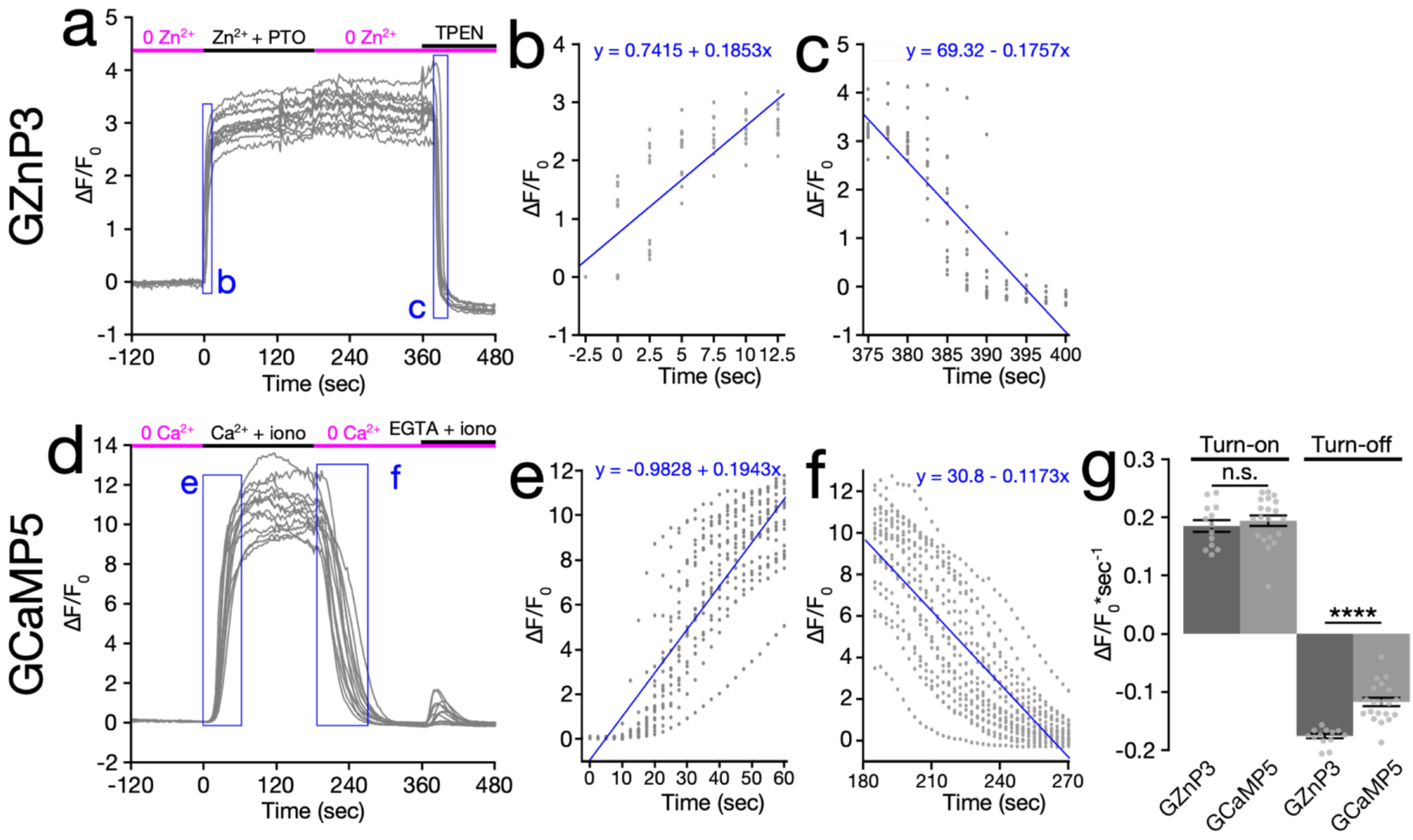

Supplementary Figure 11: ***in situ comparison of GZnP3 and GCaMP5 kinetics.***
(a) Representative traces of HeLa cells expressing GZnP3, treated with 10 µM $ZnCl_2$ and 2.5 µM pyrithione (PTO) at 0 seconds. Cells were treated with 100 µM TPEN at 360 seconds. Boxed areas indicate regions analyzed in (b) and (c). **(b)** Linear regression for GZnP3 turn on rate between -2.5 and 12.5 seconds, line of best fit and corresponding equation shown in blue. **(c)** Linear regression for GZnP3 turn off rate between 375 and 400 seconds, line of best fit and corresponding equation shown in blue. **(d)** Representative traces of HeLa cells expressing GCaMP5, treated with 1 mM $CaCl_2$ and 5 µM ionomycin (iono) at 0 seconds. Cells were treated with 5 mM EGTA and 5 µM ionomycin at 360 seconds. Boxed areas indicate regions analyzed in (e) and (f). **(e)** Linear regression for GCaMP5 turn on rate between -2.5 and 60 seconds, line of best fit and corresponding equation shown in blue. **(f)** Linear regression was run for GCaMP5 turn off rate between 185 and 270 seconds, line of best fit and corresponding equation shown in blue. **(g)** Mean slope of linear regression equations (± SEM) for GZnP3 (dark, n = 13 cells) and GCaMP5 (light, n = 20 cells) influx and efflux kinetics. Two-tailed Student's t tests. **** $p < 0.0001$. n.s., not significant. Source data are provided as a Source Data file.

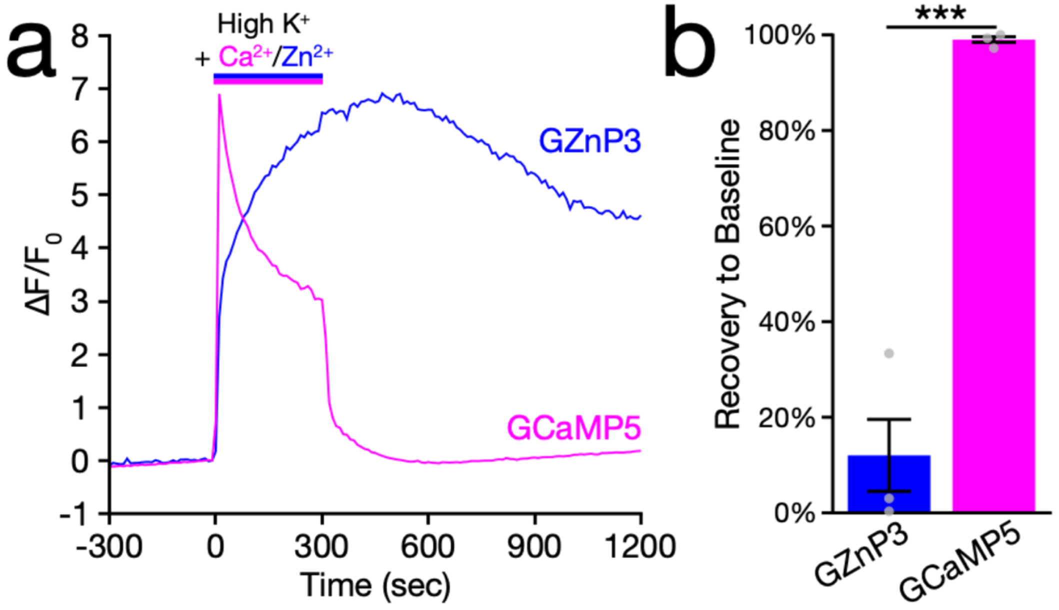

Supplementary Figure 12: ***Ca^{2+} and Zn^{2+} recovery after influx by high K^{+} depolarization.***
(a) Representative traces of neurons expressing GZnP3 (blue) or GCaMP5 (magenta). Neurons were depolarized with 50 mM KCl and 100 µM $ZnCl_2$ (blue bar) or 1 mM $CaCl_2$ (magenta bar) at 0 seconds for 5 minutes, then incubated in 0 Ca^{2+}-0 Zn^{2+} buffer for 15 minutes. **(b)** Mean percent recovery (± SEM) from maximum intensity to baseline ($\Delta F/(F_{max}-F_0)$) 15 minutes after depolarization-induced cation influx for GZnP3 and GCaMP5 (n = 3 neurons each). One-tailed Student's t test. *** $p < 0.001$. Source data are provided as a Source Data file.
